# Unusual polymorphism in new bent-shaped liquid crystals based on biphenyl as a central molecular core

**DOI:** 10.3762/bjoc.10.75

**Published:** 2014-04-07

**Authors:** Anna Kovářová, Svatopluk Světlík, Václav Kozmík, Jiří Svoboda, Vladimíra Novotná, Damian Pociecha, Ewa Gorecka, Natalia Podoliak

**Affiliations:** 1 Department of Organic Chemistry, Institute of Chemical Technology, CZ-166 28 Prague 6, Czech Republic. Fax: +420220444288; Tel: +420220444182; 2 Institute of Physics, Academy of Science of the Czech Republic, Na Slovance 2, CZ-182 21 Prague 8, Czech Republic; 3 Laboratory of Dielectrics and Magnetics, Chemistry Department, Warsaw University, Al. Zwirki i Wigury 101, 02-089 Warsaw, Poland

**Keywords:** bent-shaped, biphenyl core, liquid crystals, mesomorphic behaviour, SmC_G_ phase

## Abstract

Bent-shaped mesogens possessing a biphenyl as a central core have been synthesized and the role of the terminal chain and the orientation of the ester as a linkage group have been investigated. For the studied molecular core we have established that both parameters play an important role for the mesomorphic properties. The polyfluoroalkyl terminal chain supports the formation of mesophases, and the introduction of a chiral lactate terminal chain destabilizes mesophases for the first type of mutual orientation of ester groups, attached to the central core. On the contrary, for the opposite orientation of esters, the terminal chain has no effect on the mesomorphic properties, and columnar phases have been found for all compounds. A unique phase sequence has been found for the mesogen with the fluorinated chain. A generalized tilted smectics, SmC_G_, have been observed in a temperature interval between two different lamellar SmCP phases and characterized by X-ray and dielectric measurements. The dielectric spectroscopy data are unique and presented for the first time in the SmC_G_ phase providing new information about the molecular dynamics.

## Introduction

Achiral bent-shaped liquid crystalline (LC) compounds have attracted broad interest in the past years due to their ability to form polar mesophases [1]. From the extensive studies, molecular structure–mesomorphic property correlation have been generalized and summarized in several reviews [2–7]. The structure of bent-shaped LC materials is most commonly based on a 1,3-disubstitued benzene and a 2,7-disubstitued naphthalene central unit to which flexible lengthening arms are joined to create mesogens with symmetrical or non-symmetrical molecular architecture. Furthermore, already in the beginning of the extensive research of bent-shaped materials, biphenyl-3',4-diol, an inherently non-symmetrical structural motif, was introduced in the design of bent compounds [8]. This central unit was then utilized for synthesis and physical studies of a great deal of five- and six-ring materials exhibiting diverse mesomorphic behavior. The structural variations involved lateral substitution of the biphenyl core [9–11], lateral substitution of the outer benzene ring [12], substitution of the outer benzene for thiophene in the lengthening arms [13], introduction of siloxane [11,14–15], carbosilane [16–19], semifluorinated alkyl chains [14,20], and fullerene [21] into the terminal chain(s), variation of the linkage groups [10,22–24] (ester, azo, azoxy, imine, H-bond, cinnamoyl). Also dimeric, dendritic, and polymeric liquid crystals possessing the biphenyl moiety in the centre of the bent mesogenic unit were studied [9–11,16,25–27]. Recently, the concept of non-symmetrical bent-shaped materials based on a central hydroxyarenecarboxylic acid unit was also applied to 4'-hydroxybiphenyl-3-carboxylic acid [11,28–31] and 3'-hydroxybiphenyl-4-carboxylic acid derivatives [32]. In a study of cyano end-capped bent-shaped materials it was documented [29–31] that reversing the position of hydroxylic and carboxylic groups exerts a profound effect on the mesophase properties.

The bent-shaped molecules can create polar mesophases in spite of lack of molecular chirality. The most frequently investigated are tilted lamellar SmCP phases (B_2_ phases), in which the bent-shaped molecules are organized into polar layers with short-range in-plane positional correlations. Tilted, polar layers can be stacked in ferroelectric manner (SmC_A_P_F_ and SmC_S_P_F_ phases with a lower index F standing beneath P) or in antiferroelectric manner (SmC_S_P_A_ and SmC_A_P_A_ phases with a lower index A standing beneath P) and exhibit the synclinic (a lower index S standing beneath C) or anticlinic (a lower index A standing beneath C) molecular tilt in neighboring layers [1,33–34]. The structural chirality of subsequent layers, resulting from combination of tilt and polar order, can be the same in neighboring layers forming the homochiral phase or alternate creating a racemic state. In the bent-shaped molecular systems both the polarization and tilt are proper order parameters and they can appear independently. Existence of the polarization without molecular tilt has been proved for several materials [35–38], for which a polar orthogonal smectic A (SmAP) phase has been reported.

Additionally to the lamellar phases several two dimensionally (2D) ordered phases of bent-core molecules exist; a columnar structure can be described as a ripple phase with layer fragments forming molecular blocks. Taking into account different competing parameters as tilt angle, polarization vector and the density modulation, several columnar phases can be defined. For the structure with the density modulation plane perpendicular to the polarization vector, the nomenclature of B_1Rev_ phase was proposed [39–41]. For SmCP phases, in an applied electric field the rotation of molecules around the tilt cone is preferred. The optical switching of the columnar B_1Rev_ type of phases is very complex and the rotation around the long molecular axis with change of the structural chirality is detected more often [39–41].

Bent-shaped molecules rarely form a lamellar phase with a triclinic symmetry *C*_1_ called general tilt smectics, SmC_G_ [42–45]. The molecules in the SmC_G_ phase are tilted in such a way that the polarization vector has its component along the layer normal. Two inclination angles have to be introduced to describe the structure: the tilt of the long molecular axis from the layer normal (clinicity) and the inclination of the polarization vector from the smectic plane (leaning) [44]. This double tilt gives rise to additional freedom for building the bilayer structure–polarization vectors in consecutive layers can be synleaning or antileaning. Only few experimental studies have been reported regarding the realization of the SmC_G_ phase in bent-shaped molecules [12].

From the above mentioned it follows that bent-shaped mesogens exhibit a rich polymorphism. Moreover, also reentrancy phenomenon has been observed, which means a less ordered phase appears below a more ordered one on cooling. The reentrancy of the SmCP_A_ phase below an oblique columnar phase has been observed for 4-chlororesorcinol bent-shaped compounds [46].

In this paper we present results obtained for new bent-shaped mesogens based on thehydroxybiphenylcarboxylic core. We emphasize the complexity of the mesophase behavior and present a new phase sequence with a reentrancy of the SmCP phase. Additionally, the 2D modulated phase with local ‘general tilt’ structure has been proved and characterized in this phase sequence by various experimental methods.

## Results and Discussion

### Synthesis of the target compounds

**Figure 1:**
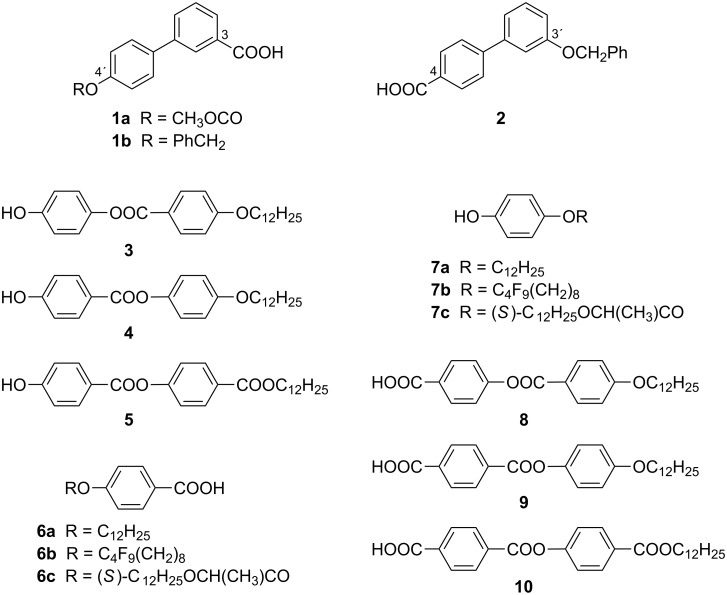
Structure of the central cores and lengthening arms.

For the synthesis of the isomeric materials **I**–**VI**, we utilized the standard three-step acylation/deprotection/acylation process of joining the lengthening arms to the central unit represented by the protected acid **1a,b** and **2**, respectively (Figure 1). The hydroxy group in the acid **1a** was primarily protected with the methoxycarbonyl group, however, during the construction of the target materials turned out that the stability of this protecting group is not compatible with the applied reaction conditions, thus, we switched to the benzyl protection of the hydroxy group (acid **1b** and **2**).

The requested lengthening arms for the synthesis of materials of series **I**–**III** involved two-ring phenols **3**–**5** and substituted benzoic acids **6a**–**c** (Figure 1). For the preparation of series of materials **IV**–**VI**, we utilized lengthening arms of phenols **7a**–**c** and two-ring acids **8**–**10**. All intermediates were obtained previously by known methods [47–49].

**Scheme 1:**
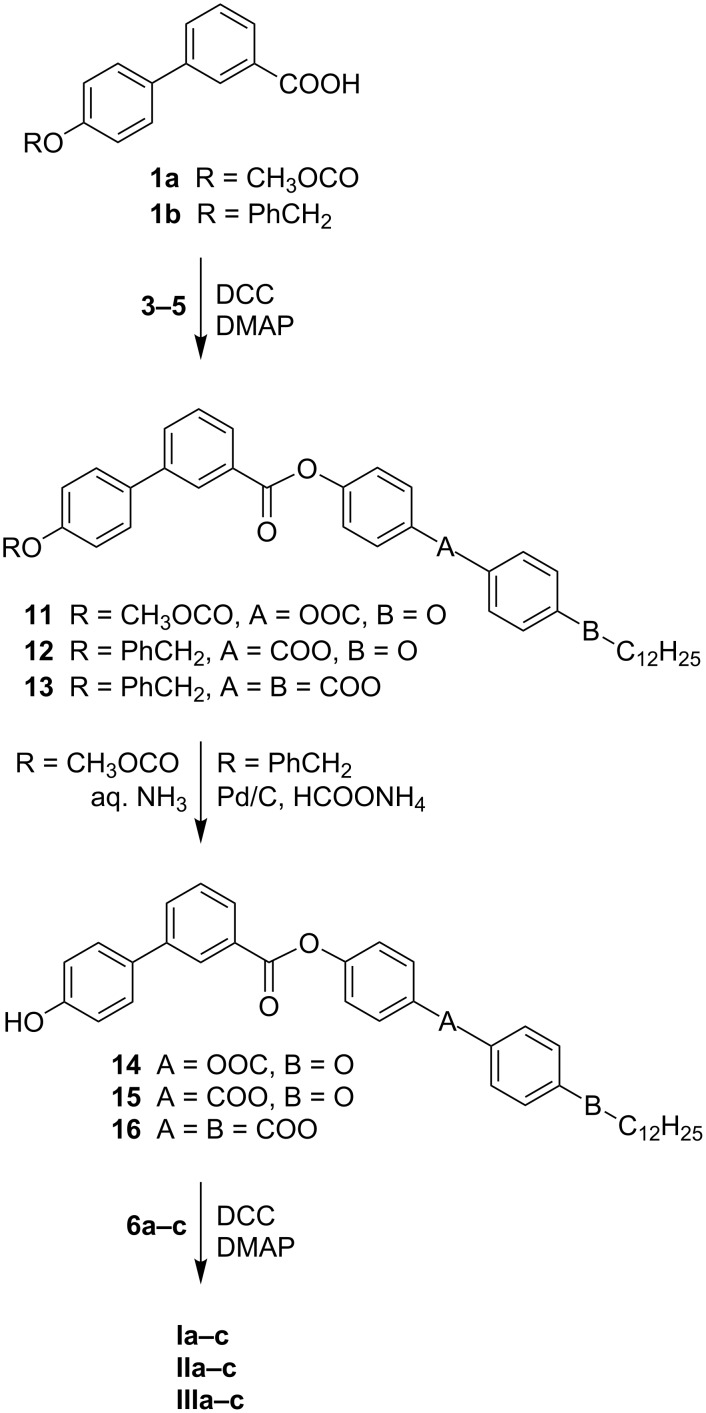
Synthesis of compounds of series **I**–**III**.

The bent-shaped liquid crystals **I**–**III** were synthesized in three steps (Scheme 1). The protected acids **1a,b** were esterified with phenols **3**–**5** in the presence of *N*,*N*'-dicyclohexylcarbodiimide (DCC) and 4-dimethylaminopyridine (DMAP) to yield esters **11**–**13** (Scheme 1). The methoxycarbonyl group in **11** was subsequently removed by means of aq. ammonia and the benzyl group in **12** and **13** by a palladium-catalysed transfer-hydrogenation. The intermediate hydroxy esters **14**–**16** were finally acylated with acids **6a**–**c** utilizing the DCC/DMAP method to yield the target compounds of series **I**–**III**.

**Scheme 2:**
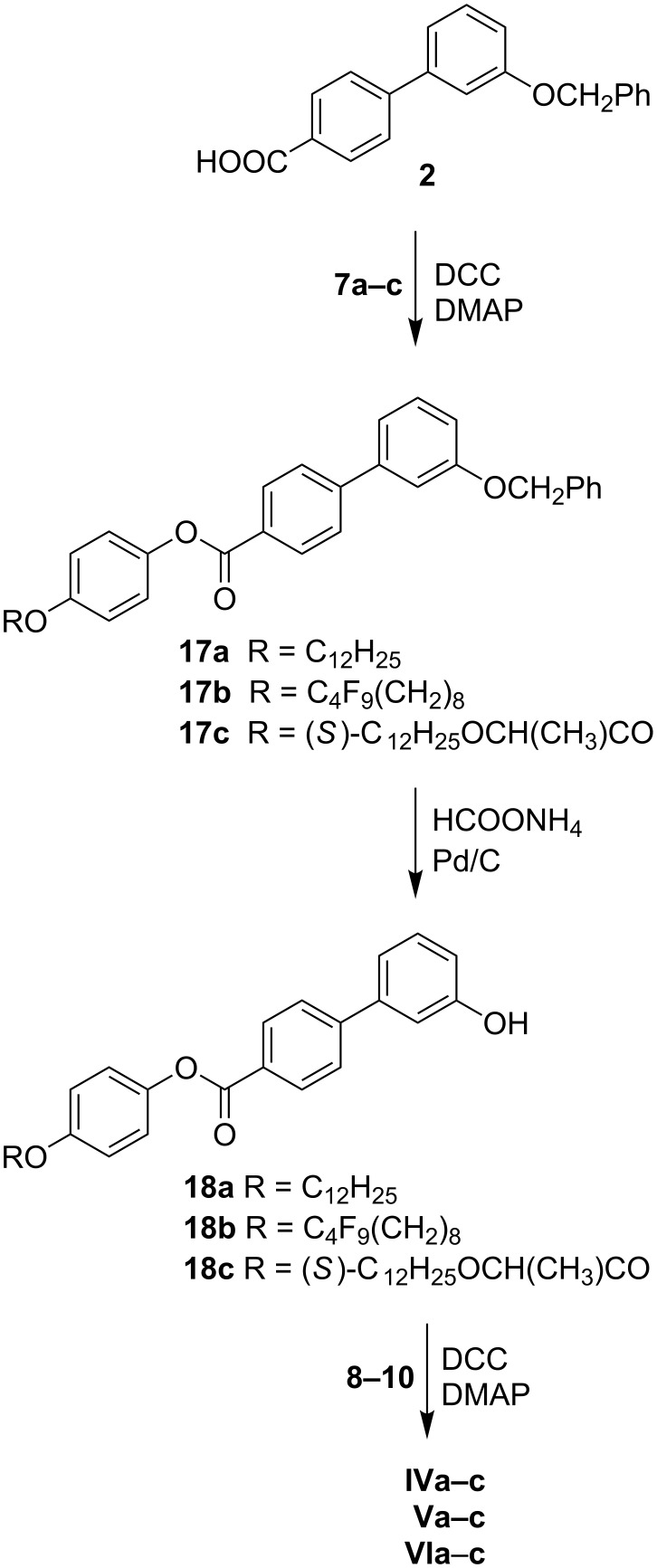
Synthesis of compounds of series **IV**–**VI**.

Series of materials **IV**–**VI** were obtained in a very similar manner starting with acid **2** (Scheme 2). In the first step, acid **2** was coupled (DCC/DMAP) with phenols **7a**–**c** to yield the phenyl esters **17a**–**c**. In the next step, the hydroxylic group was released by the palladium-catalysed hydrogenolysis of the benzyl group and the formed phenols **18a**–**c** were acylated with acids **8**–**10** to produce the series of compounds **IV**–**VI**.

**Figure 2:**
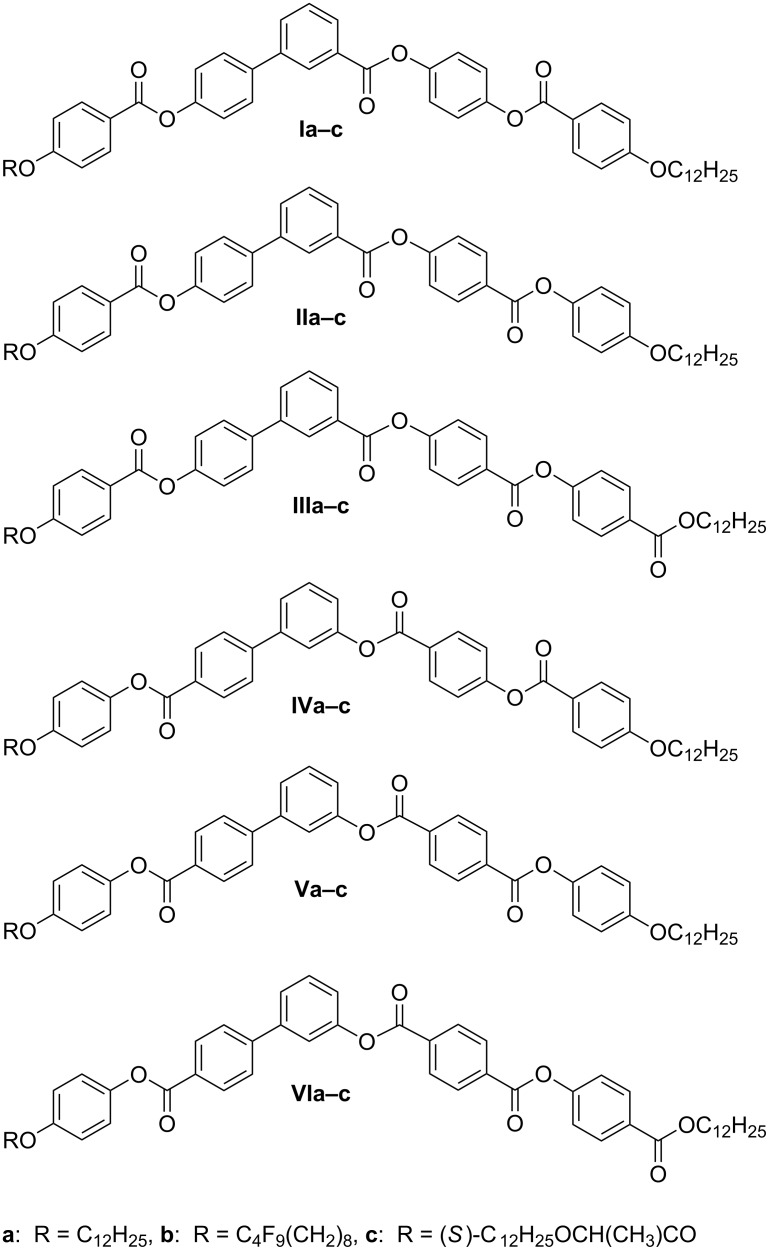
Chemical formulae of studied compounds **I**–**VI**.

For clarity, chemical formulae of the studied compounds **I**–**VI** are summarized in Figure 2.

A detailed description of synthetic procedures and characterization of all new intermediates and target compounds is summarized in Supporting Information File 1.

All the compounds were studied by DSC, Table 1 summarizes the transition temperatures and associated enthalpy changes. The data are without electric field, the electro-optic measurements are discussed separately.

**Table 1:** Melting point, mp., phase-transition temperatures, *T*_tr_, in °C, and corresponding enthalpies, Δ*H*, in kJ mol^–1^ (in parenthesis) are obtained from the DSC measurements. The mp was detected in the second heating and *T*_tr_ in the second cooling run at a rate 5 K/min. Monotropic phases are presented in square brackets. LC mesophases and their identification are described in the text.

Comp.	mp (Δ*H*)	*T*_cr_ (Δ*H*)	M_4_	*T*_tr4_ (Δ*H*)	M_3_	*T*_tr3_ (Δ*H*)	M_2_	*T*_tr2_ (Δ*H*)	M_1_	*T*_tr1_ (Δ*H*)	Iso

**Ia**	120 (+38.9)	116 (−35.0)	–	–	–	–	–	–	[SmC_S_P_A_]	117 (−2.1)	•
**Ib**	117 (+29.8)	107 (−30.2)	–	–	–	–	–	–	[SmC_S_P_A_]	140 (−17.2)	•
**Ic**	110 (+30.2)	101 (−28.7)	–	–	–	–	–	–	[SmC_S_P_A_]	105 (−10.1)	•

**IIa**	113 (+48.6)	112 (−11.9)	–	–	–	–	–	–	B_1Rev_	138 (−19.2)	•
**IIb**	105 (+32.0)	89 (−19.4)	SmC_S_P_A_	138 (−0.2)	SmC_G_	148 (−0.1)	SmC_A_P_A_	154 (−0.1)	SmAP	157 (−15.4)	•
**IIc**	119 (+87.7)	115 (−77.0)	–	–	–	–	–	–	–	–	•

**IIIa**	116 (+39.3)	111 (−35.4)	–	–	–	–	–	–	B_1Rev_	137 (−18.0)	•
**IIIb**	100 (+20.2)	95 (−16.1)	–	–	–	–	B_1Rev_	148 (−1.2)	SmAP	155 (−13.0)	•
**IIIc**	103 (+63.0)	96 (−37.8)	–	–	–	–	–	–	–	–	•

**IVa**	136 (+63.5)	116 (−37.0)	–	–	–	–	–	–	[B_1Rev_]	125 (−20.8)	•
**IVb**	129 (+34.8)	110 (−30.7)	–	–	–	–	–	–	B_1Rev_	154 (−23.7)	•
**IVc**	107 (+33.7)	96 (−29.2)	–	–	–	–	–	–	B_1Rev_	143 (−20.8)	•

**Va**	145 (+62.6)	119 (−43.6)	–	–	–	–		–	[B_1Rev_]	144 (−18.8)	•
**Vb**	133 (+39.3)	113 (−36.3)	–	–	–	–	B_1Rev’_	159 (−0.2)	B_1Rev_	166 (−19.3)	•
**Vc**	136 (+63.5)	116 (−37.0)	–	–	–	–	B_1Rev’_	152 (−0.6)	B_1Rev_	157 (−16.9)	•

**VIa**	101 (+29.0)	84 (−24.7)	–	–	–	–	–	–	B_1Rev_	112 (−18.7)	•
**VIb**	120 (+23.1)	96 (−24.0)	–	–	–	–	–	–	B_1Rev_	129 (−14.0)	•
**VIc**	118 (+39.7)	92 (−27.3)	–	–	–	–	–	–	[B_1Rev_]	105 (−15.7)	•

**Figure 3:**
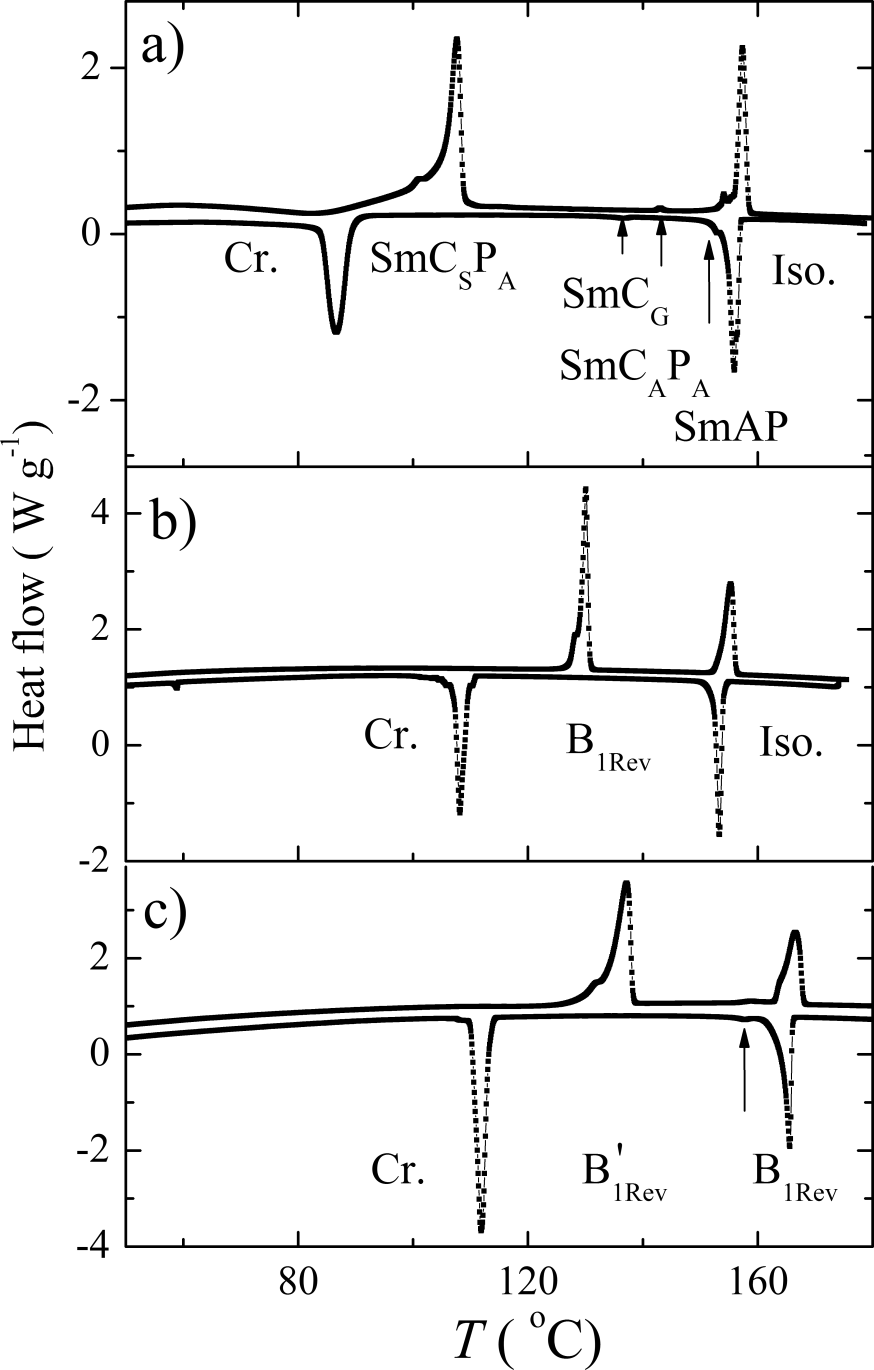
DSC plots for compounds a) **IIb,** b) **IVb** and c) **Vb** taken on second heating (upper curve) and cooling (lower curve) at a rate of 5 K min^–1^. Mesophases are designated, arrows mark the phase transitions.

DSC plots for three selected compounds **IIb**, **IVb** and **Vb**, taken on second heating and cooling runs are demonstrated in Figure 3. As it was reported for other bent-shaped materials [4,47–49], the type of mesophase depends on the orientation of the ester linkage group as well as on the length and character of the terminal chains.

All compounds of series **I** exhibit the SmC_A_P_A_ phase with typical textures and behavior in the electric field. Reversing the orientation of one ester group (materials **IIa**–**c**) dramatically changes the character of the formed mesophases. While the material **IIa** exhibits only the B_1_ phase, rich polymorphism was found for its polyfluoroalkyl substituted analogue **IIb**, SmAP–SmC_A_P_A_–SmC_G_–SmC_S_P_A_ phase sequence on cooling from the isotropic phase was observed. For materials of series **III**, the columnar B_1Rev_ phase appears for **IIIa** and the sequence SmAP-B_1Rev_ was found in compound **IIIb**. The materials **IIc** and **IIIc** possessing the chiral lactate unit in the terminal chain were not mesogenic. In series **IV**–**VI** the orientation of the ester group attached to the biphenyl unit is reversed in comparison with series **I**–**III**. All materials **IV**–**VI** form the columnar B_1Rev_ type of phase. For compounds **Vb** and **Vc**, a sequence of two different B_1Rev_ phases has been detected on cooling from the isotropic phase. The character of mesophase has been confirmed by X-ray and other experimental techniques and will be described in details later.

### Series I

**Figure 4:**
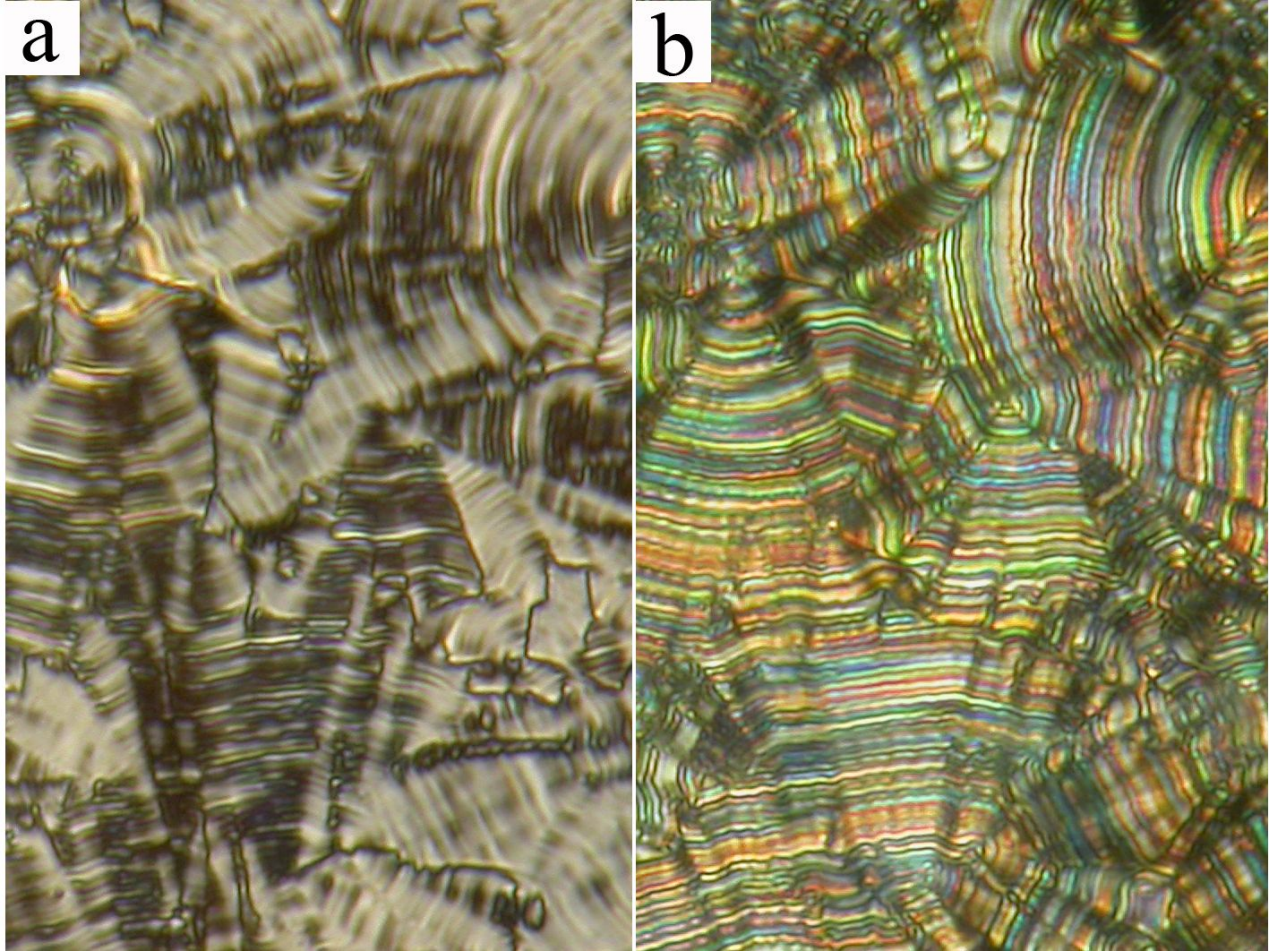
Planar texture of **Ib** in the SmC_A_P_A_ phase at temperature *T* = 130 °C (a) without field, and (b) in the applied electric field 25 V/μm, when the system turns to the SmC_S_P_A_ phase. The width of the microphotograph is 250 μm.

Compounds **Ia** and **Ic** exhibit the SmC_A_P_A_ phase in a very narrow temperature interval on cooling only (in both materials the smectic phase is monotropic). Introduction of a perfluoroalkyl chain in material **Ib** has a pronounced effect on this phase, which is stabilized in a rather broad temperature range between 140 °C and 117 °C. Unfortunately **Ia** and **Ic** often crystallized under electric field, so we will demonstrate the physical behavior of the SmC_A_P_A_ phase for **Ib**. We have observed the textures under a polarizing microscope with crossed polarizer and analyser orientation. On cooling from the isotropic phase the leaf-like texture was grown with coloured features. When we applied a DC electric field, typical fan-shaped texture appears. Under the electric field the ferroelectric layers grow at the expense of the antiferroelectric ones [50]. The stripe texture persists up to the maximum of the applied electric field 25 V/μm evidencing the presence of the separated ferroelectric domains of opposite chirality. After switching off the electric field, the fan-shape texture darkens indicating the low birefringence and the extinction positions coincide with the polarizers direction. This type of texture can be identified with a SmC_A_P_A_ phase. We can suppose that the SmC_A_P_A_–SmC_S_P_F_ phase transformation takes place under the applied electric field (Figure 4).

Switching in a low-frequency electric field with an AC electric field with the triangular profile is accompanied by two current peaks per a half cycle. X-ray diffraction measurements have been performed for **Ib** to confirm the lamellar character of the SmC_A_P_A_ phase. The layer spacing, *d*, has been found to decrease slightly on cooling from 40.4 Å at *T* = 135 °C to 40.0 Å at *T* = 115 °C.

### Series II

**Figure 5:**
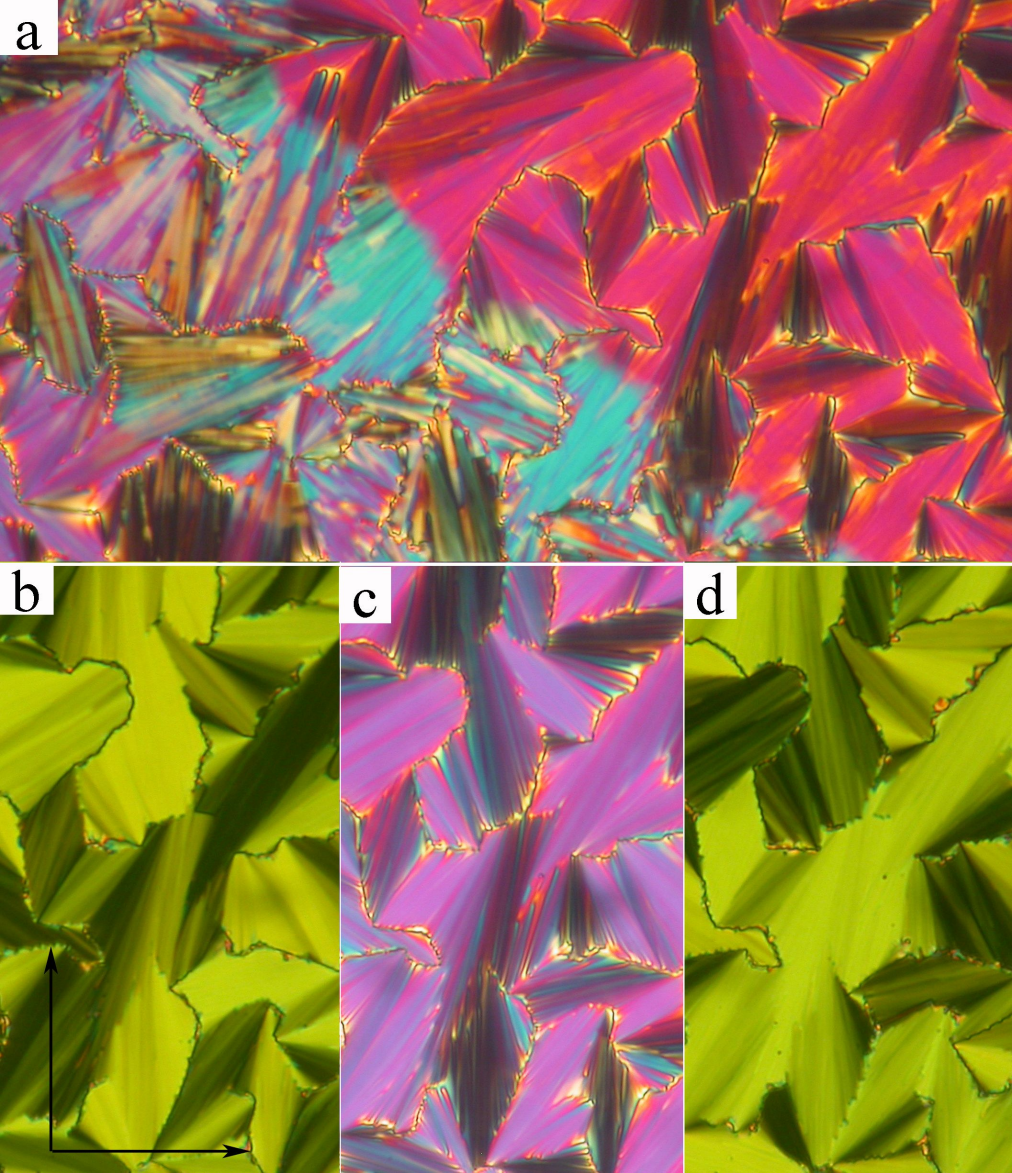
Planar texture of **IIb** (a) at the phase transition from the SmAP (upper right corner) to the SmC_A_P_A_ phase (down left corner). Planar texture in the SmC_A_P_A_ phase at temperature *T* = 130 °C in the applied electric field (b) E = +20 V/μm, (c) without field, and (d) under E = −20 V/μm. The width of the microphotograph is 200 μm. Arrows mark directions of crossed polarizers in the microscope.

For compound **IIa** the planar textures show domains characteristic for a columnar B_1Rev_-type of phase. We will present such a texture later for another compound. For compound **IIb** a variety of phases has been found. First of all, the fan-shaped texture of a SmAP phase appeared on cooling from the isotropic phase. The extinction position lies along the layer normal, which coincides with the fan symmetry axis. By shearing the sample the planar texture could be transformed into a schlieren type. In the planar texture the SmAP–SmC_A_P_A_ phase transition is accompanied with modification of the textural features and the birefringence of fans changes (Figure 5a).

**Figure 6:**
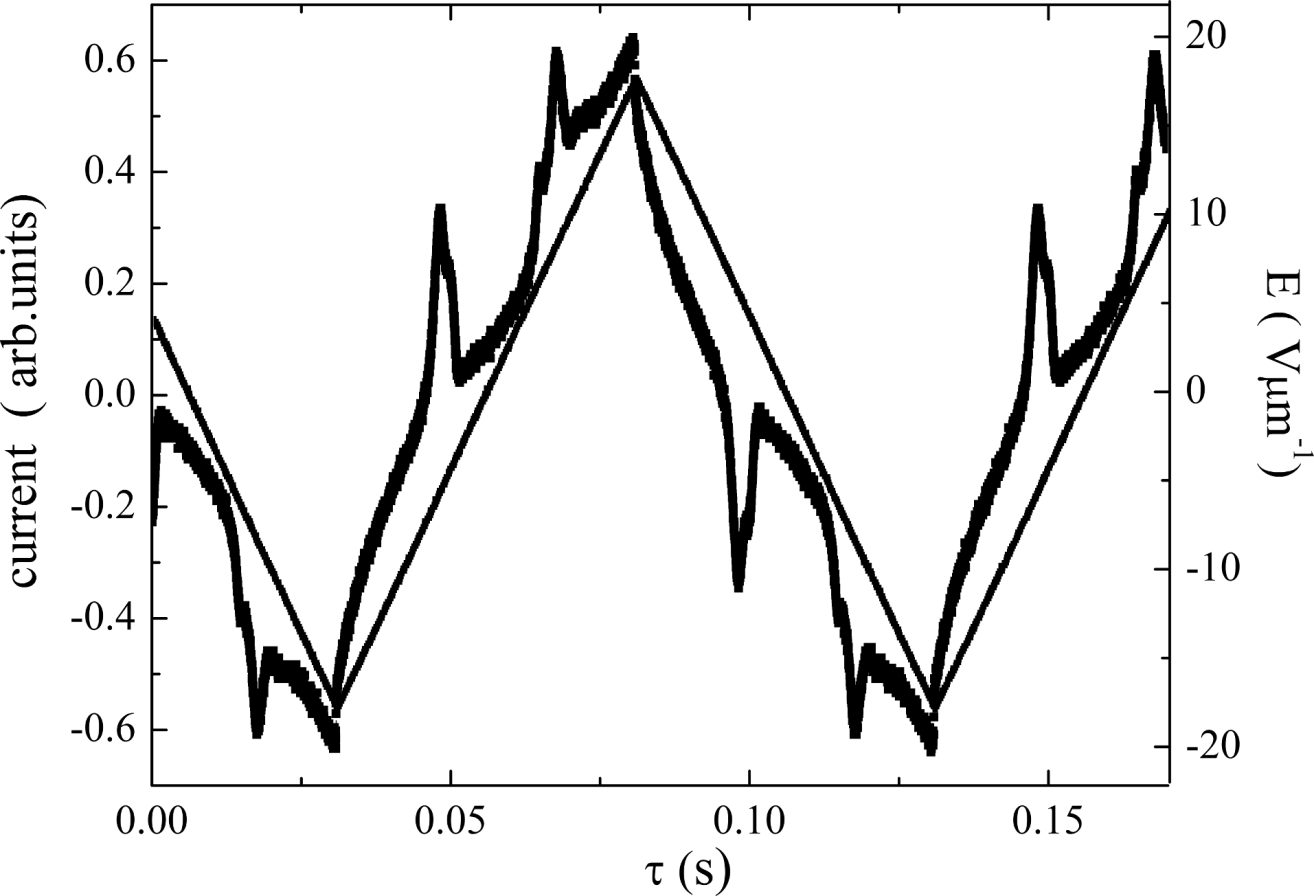
Switching current for compound **IIb** at *T* = 150 °C, taken in the SmC_A_P_A_ phase at a triangular field, *E*, at a frequency of 12 Hz.

In the SmC_A_P_A_ phase the electro-optical switching with rotation of the extinction position has been observed under an applied electric field. The texture under an applied DC electric field of +20 V/μm, and −20 V/μm is presented in Figure 5b and 5d, respectively. The applied electric field turns the extinction position clockwise and anticlockwise with respect to the texture without the field (Figure 5c). These changes are characteristic for the SmC_A_P_A_–SmC_S_P_F_ phase transformation under the field. The switching in the AC electric field of triangular profile is accompanied with two current peaks per half cycle of applied voltage (Figure 6), that supports the antiferroelectric character of the SmC_A_P_A_ phase.

**Figure 7:**
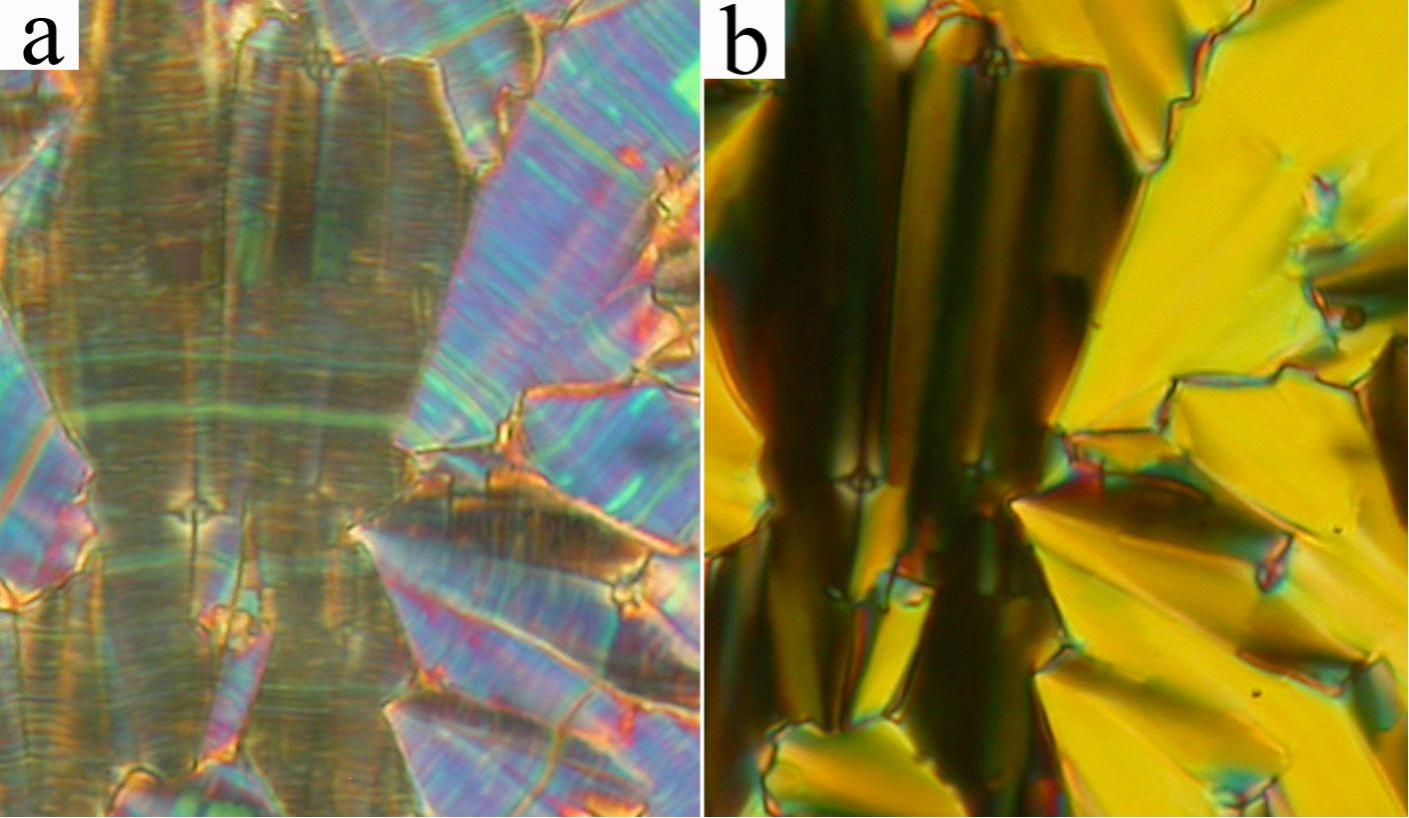
Planar texture of **IIb** compound the SmC_S_P_A_ phase at *T* = 130 °C, (a) without applied field and (b) in the applied electric field +20V/μm. The width of each microphotograph corresponds to 250 μm.

On further cooling the texture features in the planar cell slightly change at the SmC_A_P_A_–SmC_G_ phase transition at *T* = 143 °C. Application of an electric field in the SmC_G_ phase modifies the texture in such a way that one can speculate the transition from the SmC_G_ phase to the SmC_A_P_A_ phase takes place. At *T* = 138 °C another phase transition (SmC_G_–SmC_S_P_A_) is observed and the birefringence and character of the planar texture abruptly changes. In Figure 7a the SmC_S_P_A_ planar texture is presented without an electric field showing line defects parallel to the layer planes, which can be ascribed to the borderlines between synclinic domains with opposite tilt. Under the applied DC electric field, these defects disappeared (Figure 7b) and the resulting extinction position lies parallel to the layer normal evidencing the anticlinicity of the molecular arrangement in neighboring layers (the SmC_S_P_A_-SmC_A_P_F_ transition under the electric field).

**Figure 8:**
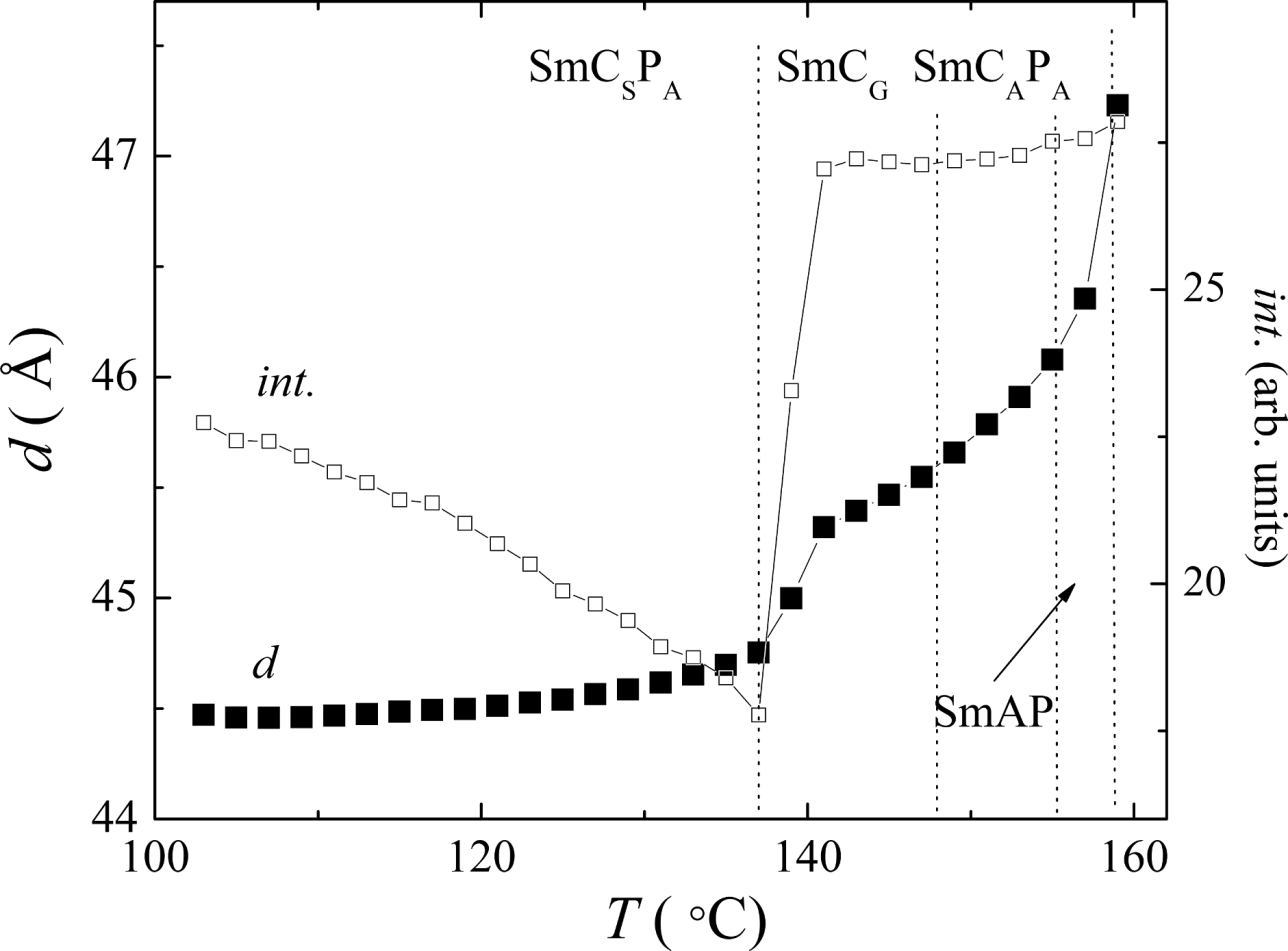
Temperature dependence of the layer spacing value, *d*, and intensity of the corresponding X-ray signal, int., for **IIb**.

**Figure 9:**
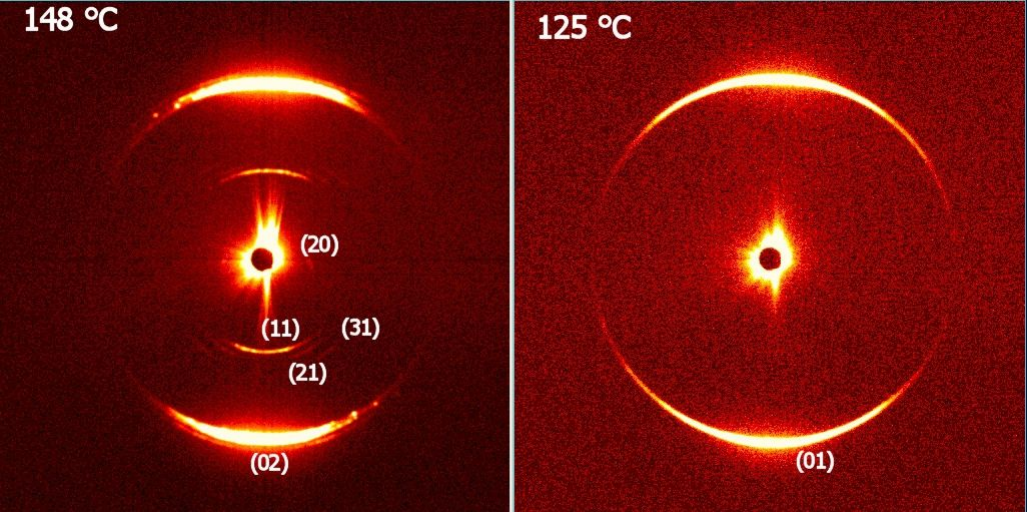
X-ray patterns of a partially aligned sample of **IIb** in (a) the SmC_G_ phase at 148 °C and (b) in the SmC_S_P_A_ phase at 125 °C. Signals in the SmC_G_ phase were indexed assuming a rectangular unit cell with *a* = 362 Å and *c* = 90.8 Å.

The X-ray measurements performed for compound **IIb** showed only commensurate signals in the small diffraction angle region for SmAP, SmC_A_P_A_ and SmC_S_P_A_ phases, reflecting the lamellar nature of the phases. The temperature dependences of the layer spacing value, *d*(T), calculated from the most intensive signal and the corresponding intensity, int., are presented in Figure 8. The intensity falls down in the vicinity of the SmC_A_P_A_–SmC_G_ and SmC_G_–SmC_S_P_A_ phase transitions. This effect can be explained with molecular fluctuation near the phase transition points. The layer spacing value decreases within the SmCP phases on cooling with respect to the *d* value of 47.1 Å in the SmAP phase, which is mostly caused by the tilt of the molecules. In the SmC_G_ phase additional signals appear in the X-ray pattern (Figure 9a) that can be indexed assuming a 2D rectangular unit cell with *a* = 362 Å and *b* = 90.8 Å. The value of parameter *b*, corresponding to a double smectic layer thickness, points to the antileaning arrangement of polarization vectors in consecutive layers, while the rectangular crystallographic unit cell evidences the anticlinic tilt structure of neighboring layers. The cross-section of molecular blocks, evaluated from the in-plane modulation periodicity a, contains about 40 molecules. For comparison the X-ray pattern of the SmC_S_P_A_ phase at 125 °C is presented in Figure 9b. Regarding the phase assignment, the x-ray patterns in the SmC_G_ phase are different from those characteristic for the B_1Rev_ phase and resemble more the typical ones for modulated lamellar phases [44,51]. It is consistent with our observations in the modulated SmC_G_ phase, in which the texture does not correspond to the B_1rev_ phase.

**Figure 10:**
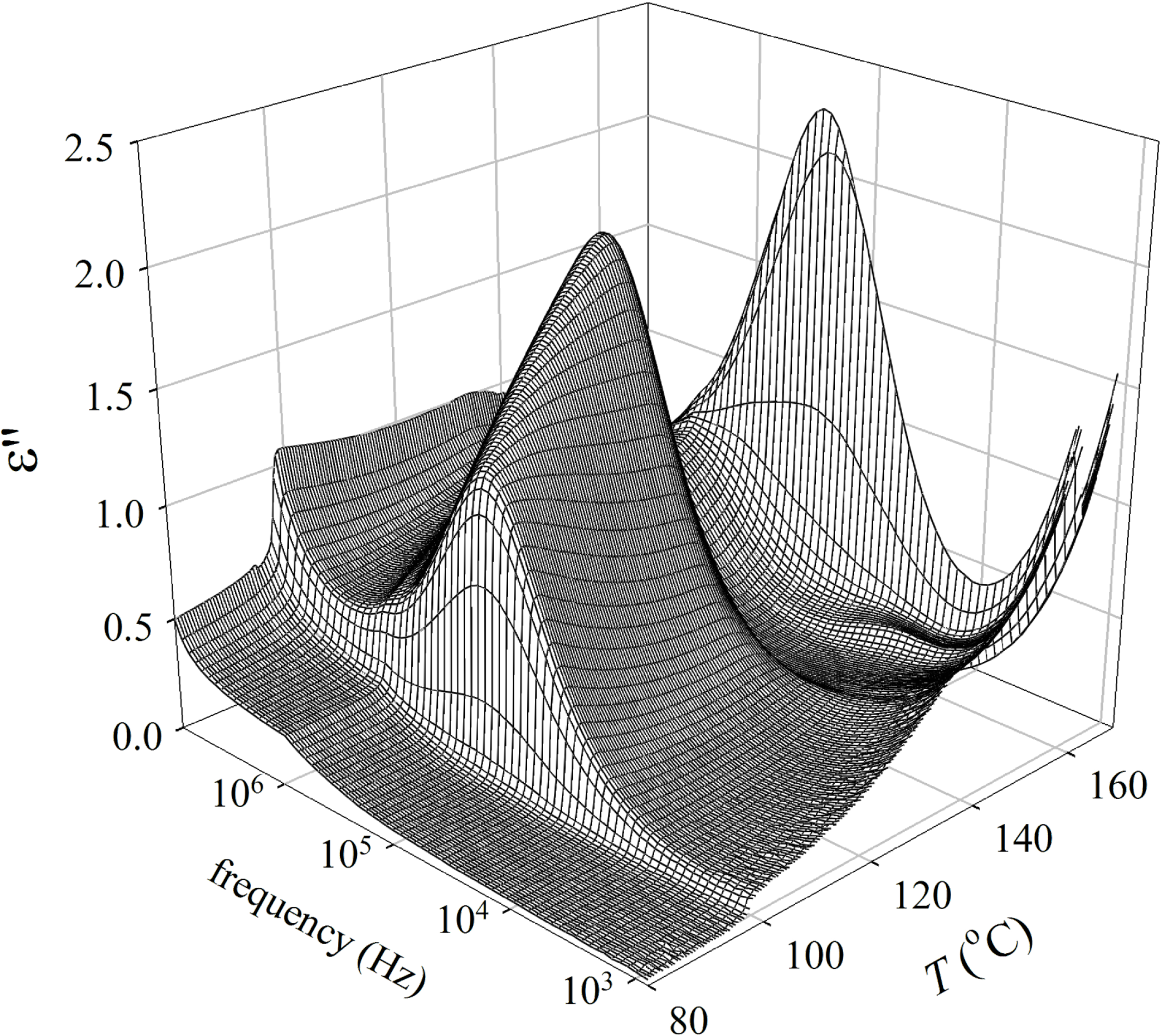
3-Dimensional plot of the imaginary part of permittivity, ε’’, versus temperature and frequency for **IIb**.

For compound **IIb** the polar fluctuations of molecules have been studied by dielectric spectroscopy. Dielectric measurements have been performed in a broad frequency and temperature range. A 3-dimensional plot of the imaginary part of the permittivity is presented in Figure 10.

**Figure 11:**
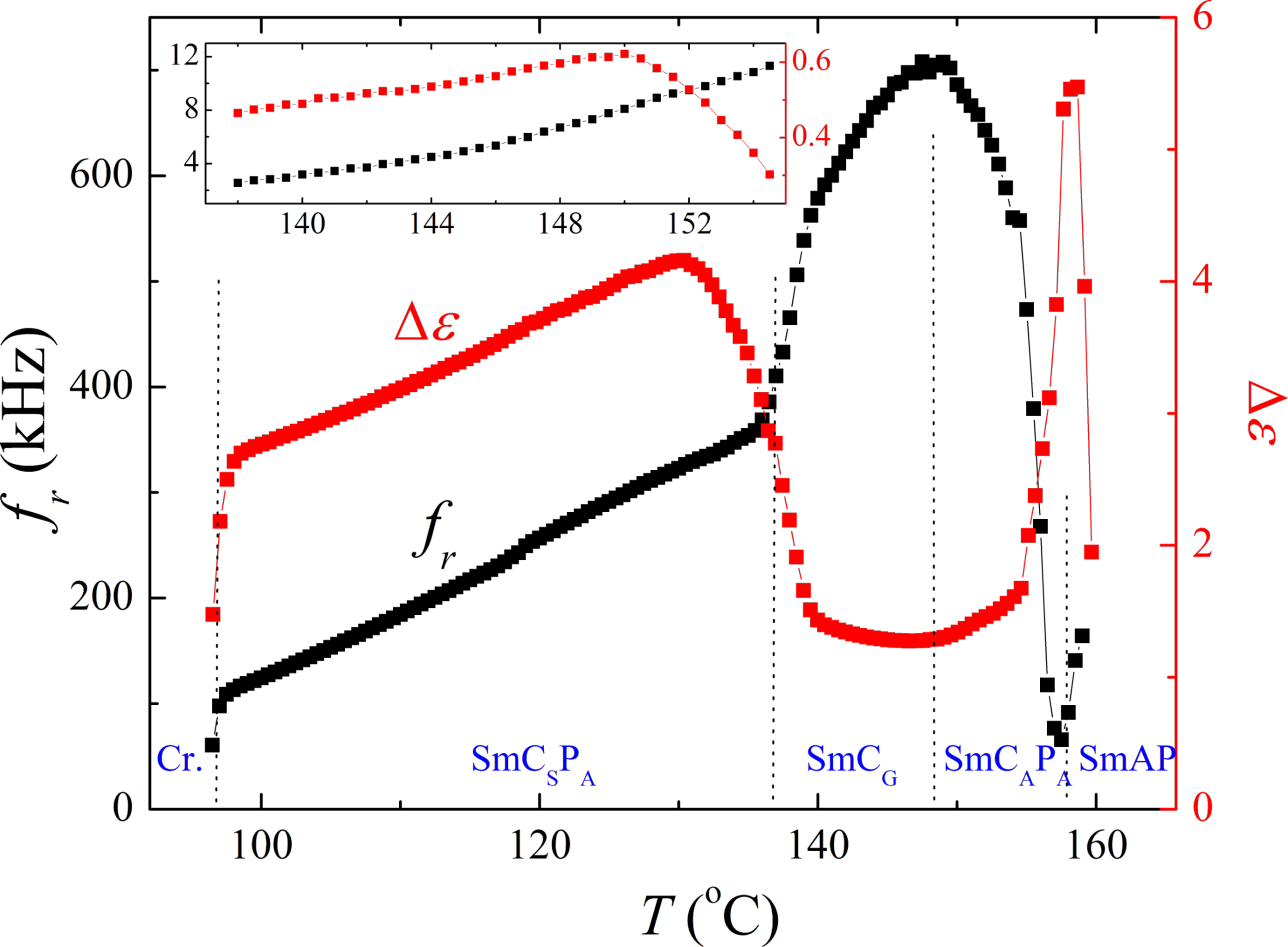
Temperature dependence of the dielectric strength, Δε, and relaxation frequency, *f_r_*, for **IIb**.

The observed mode can be attributed to collective fluctuations of molecules because it completely disappears in the isotropic as well as in the crystalline phase. Temperature dependences of the relaxation frequency, *f_r_*(*T*), and the dielectric strength, Δε(*T*), obtained by fitting to the Cole–Cole formula (Equation 1) are shown in Figure 11. The strongest fluctuations were detected in the SmAP phase and the mode strength reaches maximum value of about 5.7 at the SmAP–SmC_A_P_A_ phase transition, the corresponding relaxation frequency ~200 kHz. In the SmC_A_P_A_ phase these polar fluctuations, probably attributed to an amplitude mode, are quenched and another mode started to dominate (at a temperature *T* ~ 154 °C both modes coexisted). Besides this high frequency mode (*f_r_* ~ 500–600 kHz and Δε ≈ 1.6) a weaker mode (Δε ≈ 0.6) appeared below *T* ~ 154 °C with decreasing relaxation frequency from 12 to 4 kHz within the temperature interval of the SmC_A_P_A_ and SmC_G_ phase. At the SmC_A_P_A_–SmC_G_ phase transition, Δε of the lower frequency mode exhibits a local maximum (see the inset of Figure 11). The transition to the SmC_S_P_A_ phase is accompanied with strengthening of the upper polar mode, then both parameters Δε and *f_r_* decrease on cooling.

**Figure 12:**
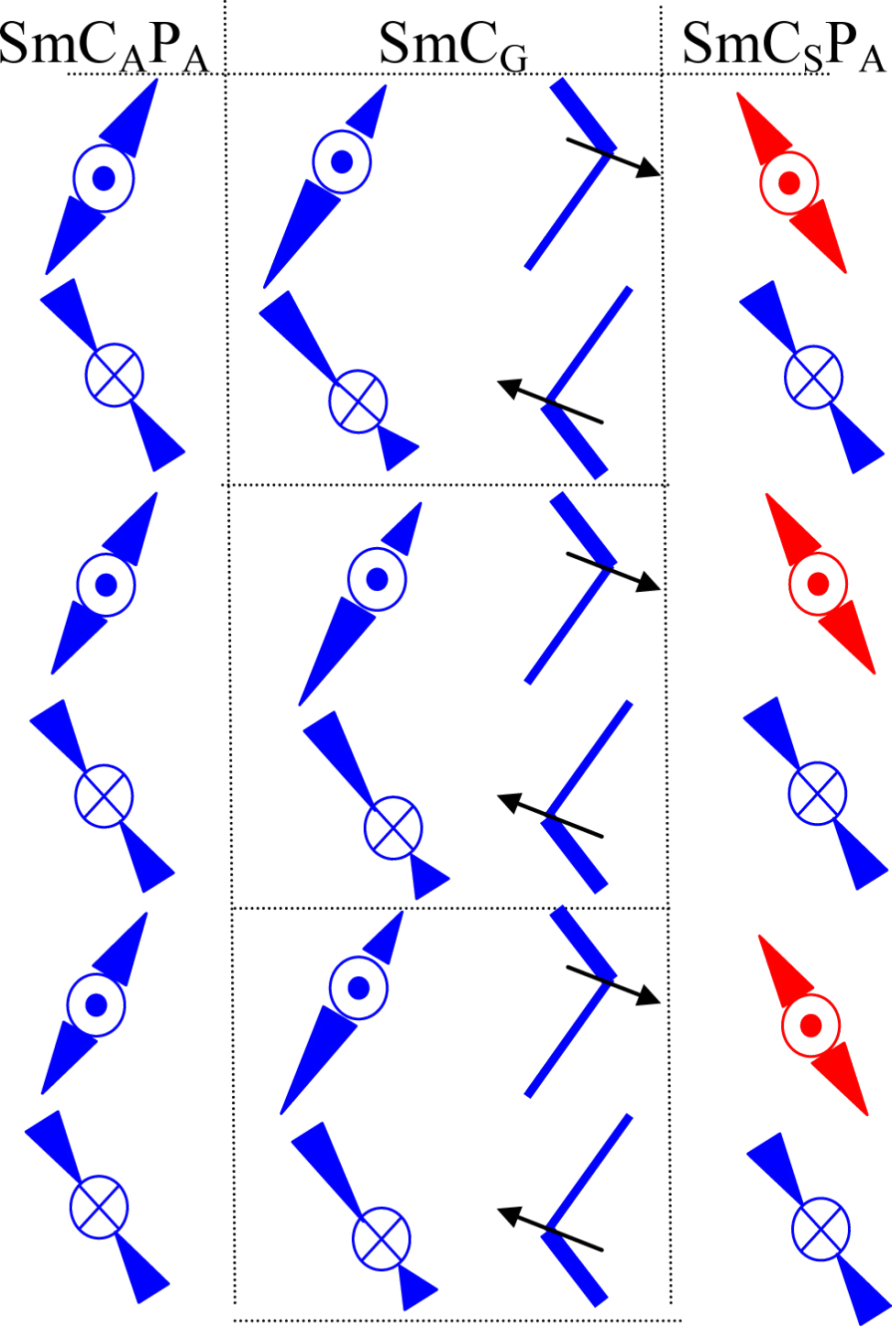
Schematic organization of bent-shaped molecules in layers for the SmC_A_P_A_–SmC_G_–SmC_S_P_A_ sequence of mesophases. In the middle, two columns show the top and side views of molecules in subsequent layers of the SmC_G_ phase.

A schematic organization of bent-shaped molecules in layers can be proposed (Figure 12). In the first column in Figure 12, the arrangement of molecules in subsequent layers of the SmC_A_P_A_ phase is shown. In the middle part, two columns show the top and side view of molecules of the SmC_G_ phase (bilayer character is pointed out). From the electro-optical and the dielectric data it follows that chirality changes at the SmC_G_ –SmC_S_P_A_ phase transition on cooling.

### Series III–VI

All compounds of series **III**–**VI**, except for non-mesogenic **IIIc**, form 2D density modulated phases of the B_1Rev_ type. For **IIIb** the SmAP–B_1Rev_ phase sequence has been found on cooling from the isotropic phase. Textures observed in the SmAP and columnar B_1Rev_ phase are presented in Supporting Information File 1. Only at the vicinity of the SmAP-B_1Rev_ phase transition an electro-optical response can be observed under polarizing microscope as a clear electro-optical switching. Out of this temperature interval the threshold voltage for the switching goes beyond the range. Two compounds of series **V** show two different columnar B_1Rev_ phases subsequently on cooling from the isotropic phase. The phase transition between these columnar phases is accompanied with a small change in planar textures, namely the birefringence changes. X-ray diffraction studies have been performed for selected compounds to confirm the phase identification and determine the structural parameters. In the SmAP phase of **IIIb**, the layer spacing value was established as 53.7 Å at *T* = 150 °C. An evaluation of the molecular length using the Chem3D software yields a value of about 54 Å, which fits to the measured layer spacing value. We can confirm the orthogonal character (without a molecular tilt with respect of the layer normal) of the SmAP phase.

X-ray profiles detected in the B_1Rev_ phases correspond to a columnar phase with oblique primitive unit cell (Table 2).

**Table 2:** Parameters of the crystallographic unit cell, measured by X-ray in the B_1Rev_ phase for several compounds at selected temperatures, *T*.

	*T* / °C	*a* / Å	*b* / Å	γ / °

**IVb**	150	174.2	47.5	91.5
	140	200.0	47.8	91.0
	130	234.3	48.2	90.8
	120	283.8	48.5	91.0
	110	370.0	48.8	91.8
**Vb**	162	142.6	48.2	96.5
	150	139.6	48.6	91.9
	140	147.6	48.8	90.7
	130	158.6	49.1	91.1
	120	171.6	49.3	91.4
**VIb**	126	130.2	48.9	95.9
	122	134.4	49.1	95.8
	118	140.1	49.3	95.6
	114	147.5	49.5	95.1
	110	156.1	49.7	94.7
	106	166.1	50.0	94.3

The strongest signal in the pattern corresponds to the molecular length, which indicates a high degree of lamellarization in the structure. The *b* parameter (reflecting roughly the length of the molecules) grows on cooling, which can be explained by stretching of the molecular tails. In all compounds, the *a* parameter, which reflects the block length, changes with temperature. There is no quantitative difference between two columnar phases B_1Rev’_ and B_1Rev_ formed by compounds **Vb** and **Vc**. The B_1Rev’_ and B_1Rev_ phase differ in the length of the molecular block (a parameter) and inclination angle of the crystallographic cell, γ.

## Conclusion

Six series **I**–**VI** of materials possessing a biphenyl as a central core were synthesized and the role of the terminal chain and orientation of the linking ester group was investigated. General questions concerned the influence of the molecular structure on the formation of polar mesophases. Both parameters (ester orientation and terminal chain character) play an important role. While the polyfluoroalkyl terminal chain supports formation of mesophases, introduction of a chiral lactate terminal chain destabilizes the mesogenic behavior (**IIc** and **IIIc** do not exhibit mesophases). For all compounds of series **IV**–**VI**, the B_1Rev_-type phases were observed and the terminal chain does not play an important role on mesogenic properties.

In material **IIb** the unique phase sequence SmAP–SmC_A_P_A_-modulated SmC_G_–SmC_S_P_A_ has been observed. The modulated phase with local triclinic symmetry, SmC_G_, has been observed between two different SmCP phases (namely the SmC_A_P_A_ and SmC_S_P_A_ phase) for the first time. The modulated SmC_G_ reveals in-plane periodicity and can be called antiphase (Figure 10). Dielectric spectroscopy and switching properties confirmed that all phases have antiferroelectric nature. The observed relaxation frequency of the dielectric response in the tilted SmCP phases points to azimuthal correlations of polarization vectors forming neighboring layers [52], i.e. the mutual rotation of molecules on the tilt cone in neighboring layers. In the modulated SmC_G_ an additional plane–density modulations exist, which has been documented by X-ray data. For generally tilted molecules, antiphason fluctuations should be strongly quenched due to the steric hindrances upon a rotation on the tilt cone. On the other hand, the synleaning fluctuations might allow only a weak dielectric response, because the change in the leaning leads to the energetically costly changes in the layer thickness. The presence of an additional polar mode in the SmC_A_P_A_ and the modulated SmC_G_ phase shows that most probably both have complex structure involving the simultaneous tilting and leaning of molecules. We can speculate that the SmC_A_P_A_ phase has also triclinic synleaning structure, although no bilayer structure is seen in X-ray measurements.

## Experimental

### Measurements

All compounds were studied using differential scanning calorimetry (DSC). The materials of 2–5 mg were hermetically closed in the aluminium pans and placed in nitrogen atmosphere of a Perkin–Elmer Pyris Diamond calorimeter. Cooling and heating rates of 5 K/min were applied. For electro-optical experiments the cells were prepared from glass plates with transparent ITO electrodes with an area of 5 × 5 mm^2^ and filled with the studied compound in the isotropic phase. The texture observations were carried out using the Nikon Eclipse polarizing microscope. The samples 3 or 6 μm thick were placed into the hot stage (Linkam) kept on the table of the polarizing microscope. The temperature was stabilized with an accuracy of ±0.1 °C. The glasses were provided with transparent ITO electrodes and polyimide layers unidirectional rubbed, which ensured the book-shelf (planar) geometry. For dielectric spectroscopy 12 μm cells with gold electrodes were used.

Dielectric properties were studied using a Schlumberger 1260 impedance analyser. The frequency dispersions were measured on cooling at a rate of about 0.2 K/min, keeping the temperature of the sample stable during the frequency sweeps in the range of 10 Hz ÷ 10 MHz. The frequency dispersion data were analysed using the Cole–Cole formula for the frequency dependent complex permittivity ε*(f) = ε′ – iε′′

[1]
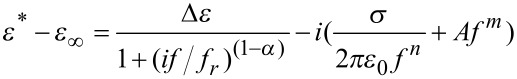


where *f*_r_ is the relaxation frequency, Δε is the dielectric strength, α is the distribution parameter of the relaxation, ε_0_ is the permittivity of a vacuum, ε_∞_ is the high frequency permittivity and n, m, A are the parameters of fitting. The terms in brackets of Equation 1 are used to eliminate a low frequency contribution from DC conductivity, σ, and a high frequency contribution due to resistance of the electrodes, respectively. Due to the gold electrodes the contribution of a parasitic term was negligible up to 2 MHz. Measured values of real, ε′, and imaginary, ε′′, part of the complex permittivity were simultaneously fitted to Equation 1.

Switching properties were studied with driving voltage from a Phillips generator PM 5191 accompanied by a linear amplifier providing a maximum amplitude of about ±120 V. A Tektronix TDC70 memory oscilloscope was used to display information about switching current profile vs. time.

X-ray diffraction studies (XRD) were performed using a Bruker Nanostar system (CuKα radiation, Vantec 2000 area detector, MRI TCPU H heating stage) working in transmission mode and a Bruker GADDS system (CuKα radiation, Vantec 2000 area detector area detector) working in reflection mode. In both systems the temperature stability was 0.1 K. Powder samples for the Nanostar system were prepared in thin-walled glass capillaries (1.5 mm diameter) and partially oriented samples for experiments in reflection were prepared as droplets on a heated surface. Molecular dimensions were estimated using the Chem3D software.

## Supporting Information

File 1 Unusual polymorphism in new bent-shaped liquid crystals with hydroxybiphenylcarboxylic acid central unit.

## References

[1] Niori T, Sekine T, Watanabe J, Furukawa T, Takezoe H (1996). J Mater Chem.

[2] Pelzl G, Diele S, Weissflog W (1999). Adv Mater.

[3] Weissflog W, Nádasi H, Dunemann U, Pelzl G, Diele S, Eremin A, Kresse H (2001). J Mater Chem.

[4] Dunemann U, Schröder M W, Amarantha Reddy R, Pelzl G, Diele S, Weissflog W (2005). J Mater Chem.

[5] Amarantha Reddy R, Tschierske C (2006). J Mater Chem.

[6] Weissflog W, Shreenivasa Murthy H N, Diele S, Pelzl G (2006). Philos Trans R Soc London, Ser A.

[7] Takezoe H, Takanishi Y (2006). Jpn J Appl Phys.

[8] Shen D, Pegenau A, Diele S, Wirth I, Tschierske C (2000). J Am Chem Soc.

[9] Dantlgraber G, Keith C, Baumeister U, Tschierske C (2007). J Mater Chem.

[10] Liao C-T, Lee J-Y, Lai C-C (2011). Mol Cryst Liq Cryst.

[11] Fergusson K M, Hird M (2010). J Mater Chem.

[12] Chen W-H, Chuang W-T, Jeng U-S, Sheu H-S, Lin H-C (2011). J Am Chem Soc.

[13] Geese K, Prehm M, Tschierske C (2010). J Mater Chem.

[14] Dantlgraber G, Eremin A, Diele S, Hauser A, Kresse H, Pelzl G, Tschierske C (2002). Angew Chem, Int Ed.

[15] Keith C, Amarantha Reddy R, Hauser A, Baumeister U, Tschierske C (2006). J Am Chem Soc.

[16] Keith C, Amarantha Reddy R, Baumeister U, Hahn H, Lang H, Tschierske C (2006). J Mater Chem.

[17] Keith C, Amarantha Reddy R, Prehm M, Baumeister U, Kresse H, Chao J L, Hahn H, Lang H, Tschierske C (2007). Chem–Eur J.

[18] Zhang Y, O'Callaghan M J, Tschierske C, Baumeister U (2008). Angew Chem, Int Ed.

[19] Zhang Y, O'Callaghan M J, Walker C, Baumeister U, Tschierske C (2010). Chem Mater.

[20] Amarantha Reddy R, Dantlgraber G, Baumeister U, Tschierske C (2006). Angew Chem, Int Ed.

[21] Vergara J, Barberá J, Serrano J L, Blanca Ros M, Sebastián N, de la Fuente R, López D O, Fernández G, Sánchez L, Martín N (2011). Angew Chem, Int Ed.

[22] Pintre I, Gimeno N, Serrano J L, Blanca Ros M, Alonso I, Folcia C L, Ortega J, Etxebarria J (2007). J Mater Chem.

[23] Liao C-T, Liu J-Y, Jiang M-H, Zhou S-F, Wu N-C, Wu Z-L, Lee J-Y (2010). Mol Cryst Liq Cryst.

[24] Gimeno N, Blanca Ros M, Serrano J L, de la Fuente M R (2004). Angew Chem, Int Ed.

[25] Kardas D, Prehm M, Baumeister U, Pociecha D, Amarantha Reddy R, Mehl G H, Tschierske C (2005). J Mater Chem.

[26] Keith C, Dantlgraber G, Amarantha Reddy R, Baumeister U, Prehm M, Hahn H, Lang H, Tschierske C (2007). J Mater Chem.

[27] Barberá J, Gimeno N, Pintre I, Blanca Ros M, Serrano J L (2006). Chem Commun.

[28] Tsvetkov N V, Ksenofontov I V, Kurakina V O, Andreeva L N, Bilibin A Yu (2002). Mol Cryst Liq Cryst.

[29] Radhika S, Sadashiva B K, Pratibha R (2010). Liq Cryst.

[30] Guo L, Dhara S, Sadashiva B K, Radhika S, Pratibha R, Shimbo Y, Araoka F, Ishikawa K, Takezoe H (2010). Phys Rev E.

[31] Gupta M, Datta S, Radhika S, Sadashiva B K, Roy A (2011). Soft Matter.

[32] Radhika S, Srinivasa H T, Sadashiva B K (2011). Liq Cryst.

[33] Link D R, Natale G, Shao R, Maclennan J E, Clark N A, Körblova E, Walba D M (1997). Science.

[34] Nakata M, Link D R, Araoka F, Thisayukta F, Takanishi Y, Ishikawa K, Watanabe J, Takezoe H (2001). Liq Cryst.

[35] Pociecha D, Gorecka E, Čepič M, Vaupotič N, Gomola K, Mieczkowski J (2005). Phys Rev E.

[36] Pociecha D, Gorecka E, Čepič M, Vaupotič N, Weissflog W (2006). Phys Rev E.

[37] Guo L, Gomola K, Gorecka E, Pociecha D, Dhara S, Araoka F, Ishikawa K, Takezoe H (2011). Soft Matter.

[38] Amarantha Reddy R, Sadashiva B K (2004). J Mater Chem.

[39] Szydlowska J, Mieczkowski J, Matraszek J, Bruce D W, Gorecka E, Pociecha D, Guillon D (2003). Phys Rev E.

[40] Gorecka E, Vaupotič N, Pociecha D, Čepič M, Mieczkowski J (2005). ChemPhysChem.

[41] Gorecka E, Vaupotič N, Pociecha D (2007). Chem Mater.

[42] Jákli A, Krüerke D, Sawade H, Heppke G (2001). Phys Rev Lett.

[43] Eremin A, Diele S, Pelzl G, Nádasi H, Weissflog W (2003). Phys Rev E.

[44] Gorecka E, Pociecha D, Vaupotič N, Čepič M, Gomola K, Mieczkowski J (2008). J Mater Chem.

[45] Eremin A, Jákli A (2013). Soft Matter.

[46] Shreenivasa Murthy H N, Bodyagin M, Diele S, Baumeister U, Pelzl G, Weissflog W (2006). J Mater Chem.

[47] Kohout M, Svoboda J, Novotná V, Pociecha D, Glogarová M, Gorecka E (2009). J Mater Chem.

[48] Kohout M, Svoboda J, Novotná V, Glogarová M, Pociecha D (2010). Liq Cryst.

[49] Kohout M, Svoboda J, Novotná V, Pociecha D (2011). Liq Cryst.

[50] Lejček L, Novotná V, Glogarová M (2011). Phys Rev E.

[51] Coleman D A, Jones C D, Nakata M, Clark N A, Walba D M, Weissflog W, Fodor-Czorba K, Watanabe J, Novotna V, Hamplova V (2008). Phys Rev E.

[52] Gorecka E, Pociecha D, Čepič M, Žekš B, Dabrowski R (2002). Phys Rev E.

