# Supplementary material for: Unusual polymorphism in new bent-shaped liquid crystals based on biphenyl as a central molecular core
Source: Beilstein J Org Chem. 2014 Apr 7;10:794–807. doi: 10.3762/bjoc.10.75 (PMC3999814; doi:10.3762/bjoc.10.75)
Supplement: File 1 — Unusual polymorphism in new bent-shaped liquid crystals with hydroxybiphenylcarboxylic acid central unit. [file Beilstein_J_Org_Chem-10-794-s001.pdf]

Supporting Information
for
Unusual polymorphism in new bent-shaped liquid crystals based on biphenyl as a central molecular core

Anna Kovářová[1], Svatopluk Světlík[1], Václav Kozmík[1], Jiří Svoboda[1*], Vladimíra Novotná[2], Damian Pociecha[3], Ewa Gorecka[3] and Natalia Podoliak[2]

Address: [1]Department of Organic Chemistry, Institute of Chemical Technology, CZ-166 28 Prague 6, Czech Republic. Fax: +420220444288; Tel: +420220444182, [2]Institute of Physics, Academy of Science of the Czech Republic, Na Slovance 2, CZ-182 21 Prague 8, Czech Republic and [3]Laboratory of Dielectrics and Magnetics, Chemistry Department, Warsaw University, Al. Zwirki i Wigury 101, 02-089 Warsaw, Poland

Email: Jiri Svoboda* – Jiri.Svoboda@vscht.cz

*Corresponding author

Unusual polymorphism in new bent-shaped liquid crystals with hydroxybiphenylcarboxylic acid central unit

1. General

^{1}H NMR spectra were recorded on Varian Gemini 300 HC instrument at 300 MHz; deuteriochloroform and hexadeuteroacetone were used as solvent, resp., and signals of the solvent served as internal standard. Chemical shifts are given in ppm and *J* values are given in Hz. Elemental analyses were carried out on Elementar vario EL III instrument. The purity of all final compounds was checked by HPLC analysis (Tessek C18 25 × 4.5 RP column) and found to be higher than 99.8%. Column chromatography was carried out using Merck Kieselgel 60 (60–100 μm).

2. Synthesis of the central biphenylcarboxylic acids and the lengthening arms

Acids **1a**,**b** and **2** representing the protected central units were obtained according to the synthetic path shown in Scheme S1. Synthesis of the 4´-protected biphenyl-3-carboxylic acid started with the Pd^0-catalyzed Suzuki cross-coupling of 4-methoxyphenylboronic acid with ethyl 3-iodobenzoate yielding ester **19**. Both the methyl and ethyl groups were split off by the means of boron tribromide in dichloromethane and the hydroxylic group in the parent acid **20** was primarily protected with the methoxycarbonyl group to form derivative **1a** or benzylation yielding the acid **1b**.

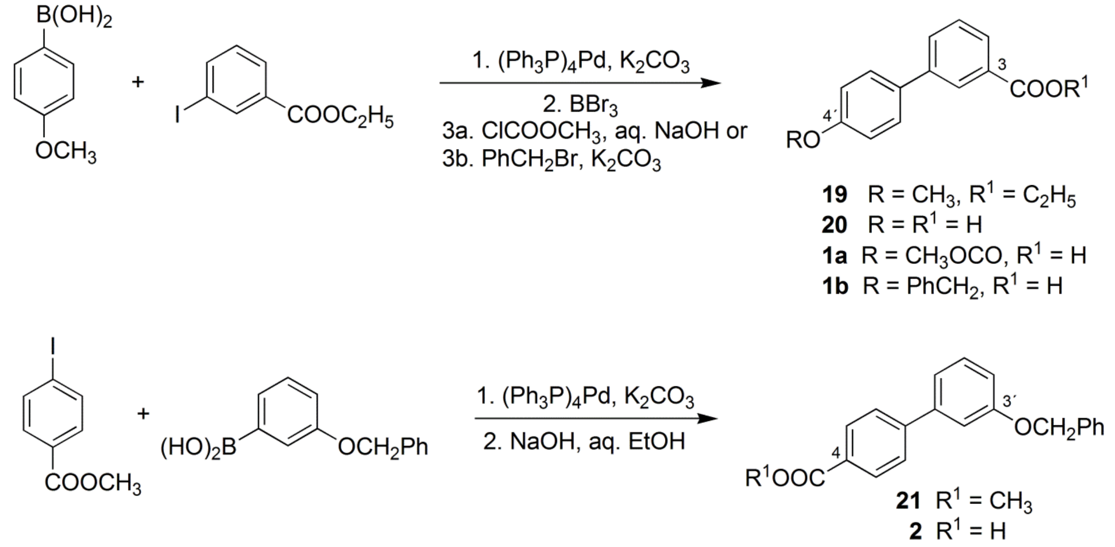

Scheme S1: Synthesis of protected acids **1a,b** and **2**.

By the same concept, the isomeric 3´-substituted biphenyl-4-carboxylic acid **2** was obtained: methyl 4-iodobenzoate was coupled with 3-benzyloxyphenylboronic acid to yield ester **21**, which was subsequently hydrolyzed to acid **2**.

The semifluorinated phenol **7b** was obtained by a multistep procedure starting with 4-benzyloxyphenol (**22**) (Scheme S2). First, the hydroxylic group was alkylated with 8-bromooct-1-ene to form the unsaturated ether **23**. In the next step, perfluorobutyl iodide was added to the terminal double bond in a radical reaction initiated with azobis(isobutyronitrile) (AIBN) and subsequently, the iodine atom in the adduct **24** was removed by reduction with $LiAlH_4$. Finally, deprotection of the benzyl group in **25** was achieved by transfer-hydrogenation yielding the phenol **7b**.

In the first step of synthesis of phenol **7c**, the *tert*-butyldimethylsilyl (TBDMS) protected hydroquinone **26** (Scheme S2) was acylated with (*S*)-dodecyloxypropanoic acid in a dicyclohexylcarbodiimide/dimethylaminopyridine (DCC/DMAP) mediated reaction and the protecting silyl group in the formed ester **27** was removed by the means of tetrabutylammonium fluoride (TBAF).

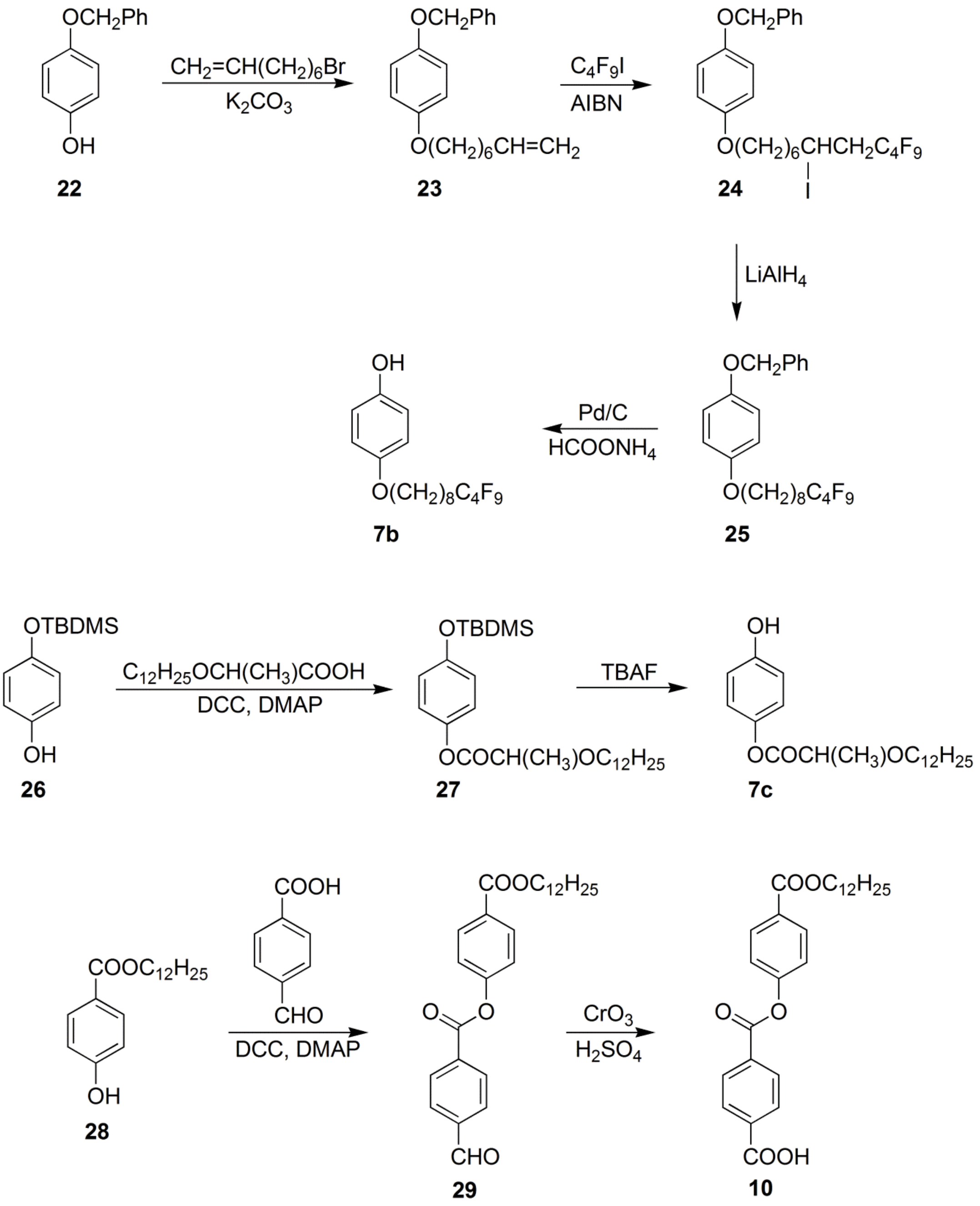

Scheme S2: Synthesis of the lengthening arms.

Acid **10** (Scheme S2) was prepared in a two-step procedure as reported previously for related compounds described in ref. [S1]. The hydroxylic group of **28** was acylated with 4-formylbenzoic acid to yield the formyl ester **29**. In the second step, the formyl group was oxidized with Jones reagent (CrO_3/H_2SO_4) to give rise to acid **10**.

Ethyl 4´-methoxy-1,1´-biphenyl-3-carboxylate (**19**)

A mixture of ethyl 3-iodobenzoate (5.0 g; 18.1 mmol), 4-methoxyphenylboronic acid (3.8 g; 25.0 mmol), tetrakis(triphenylphosphine)palladium (0.51 g; 0.48 mmol) and potassium carbonate (3.0 g; 22.0 mmol) in DMF (60 mL) was stirred and heated at 110 °C for 48 h in an argon atmosphere. After cooling to room temperature, the reaction mixture was diluted with

water (80 mL) and extracted with ethyl acetate (3 × 60 mL). The combined organic solution was washed with brine (50 mL) and dried with anhydrous magnesium sulfate. After evaporation, the product was purified by column chromatography (toluene). 4.15 g (90%) of oily ester **19** was obtained. ^{1}H NMR: 1.42 (t, 3 H, *J* = 6.4, CH_3), 3.86 (s, 3 H, OCH_3), 4.41 (q, 2 H, CH_2), 7.00 (d, 2 H, *J* = 8.5, H-3´, H-5´), 7.48 (t, 1 H, H-5), 7.57 (d, 2 H, *J* = 8.5, H-2´, H-6´), 7.74 (d, 1 H, *J* = 7.9, H-6), 7.99 (d, 1 H, *J* = 7.6, H-4), 8.25 (s, 1 H, H-2).

4´-Hydroxy-1,1´-biphenyl-3-carboxylic acid (**20**)

To a solution of ester **19** (4.1 g; 16.0 mmol) in dichloromethane (200 mL) cooled to −70 °C, boron tribromide (3.9 mL) was added drop-wise and the reaction mixture was stirred for 48 h at room temperature in an argon atmosphere. The mixture was diluted with water (150 mL) and extracted with dichloromethane (3 × 100 mL). After evaporation of the solvent, the crude product was purified by crystallisation from toluene. 3.34 g (97%) of acid **20** was obtained, m.p. 239-241 °C, ref. [S2] 241-242 °C.

4´-[(Methoxycarbonyl)oxy]-1,1´-biphenyl-3-carboxylic acid (**1a**)

To an ice cold mixture of acid **20** (1.0 g; 4.66 mmol), sodium hydroxide (0.48 g; 12 mmol), water (40 mL) and THF (40 mL), methyl chloroformate (0.58 mL; 0.71 g; 7.5 mmol) was added drop wise. After 15 min, the reaction mixture was acidified with 15% aq. hydrochloric acid to pH ~2. The solvent was evaporated and the precipitate was filtered off, washed with water (2 × 15 mL), and crystallized from ethyl acetate to yield 0.98 g (77%) of acid **1a**, m. p. 187-188 °C. ^{1}H NMR (acetone-d_6): 3.89 (s, 3 H, CH_3OCO), 7.36 (d, 2 H, *J* = 9.0, H-3´, H-5´), 7.63 (t, 1 H, H-5), 7.78 (d, 2 H, *J* = 8.8, H-2´, H-6´), 7.94 (d, 1 H, *J* = 7.6, H-6), 8.05 (d, 1 H, *J* = 7.6, H-4), 8.30 (s, 1 H, H-2). Elemental analysis: for $C_{15}H_{12}O_5$ (272.26): calculated C 66.17, H 4.44; found C 65.93, H 4.37%.

4´-(Benzyloxy)-1,1´-biphenyl-3-carboxylic acid (**1b**)

To a mixture of acid **20** (1.1 g; 5.1 mmol) and potassium carbonate (1.08 g; 7.8 mmol) in acetone (70 mL), benzyl bromide (1.86 g; 10.8 mmol) was added and the reaction mixture was stirred and heated to boiling for 8 h. After cooling to room temperature, the mixture was diluted with water (60 mL) and extracted with chloroform (3 × 50 mL) and the solvent was evaporated. The residue was diluted with ethanol (50 mL) and a solution of sodium hydroxide (8.0 g; 0.2 mol) in water (12 mL) was added. The mixture was stirred at room temperature for 3 h, cooled to 0 °C and acidified with 15% aq. HCl to pH ~2. The precipitate was filtered off, washed with water (2 × 15 mL) and crystallized from toluene. 1.2 g (77%) of acid **1b** was isolated, m. p. 221-223 °C. ^{1}H NMR (acetone-d_6): 5.20 (s, 2 H, OCH_2), 7.16 (d, 2 H, *J* = 8.8, H-3´, H-5´), 7.40 (m, 3 H), 7.52 (m, 2 H), 7.57 (t, 1 H, H-5), 7.67 (d, 2 H, *J* = 8.8, H-2´, H-6´), 7.88 (d, 1 H, *J* = 8.2, H-6), 7.99 (d, 1 H, *J* = 7.6, H-4), 8.26 (s, 1 H, H-2). Elemental analysis: for $C_{20}H_{16}O_3$ (304.35): calculated C 78.93, H 5.30; found C 78.78, H 5.15%.

Methyl 3´-benzyloxy-1,1´-biphenyl-4-carboxylate (**21**)

A mixture of methyl 4-iodobenzoate (6.7 g; 26.0 mmol), 3-benzyloxyphenylboronic acid (8.0 g; 35.0 mmol), tetrakis(triphenylphosphine)palladium (0.9 g; 0.78 mmol) and potassium carbonate (6.0 g; 43.0 mmol) in DMF (120 mL) was stirred and heated at 100 °C in an argon

atmosphere for 24 h. After cooling to room temperature, the reaction mixture was diluted with water (200 mL) and extracted with ethyl acetate (3 × 100 mL). The combined organic solution was washed with water (80 mL) and brine (80 mL) and dried with anhydrous magnesium sulfate. The crude product after evaporation was purified by crystallization from methanol. 6.50 g (80%) of ester **21** was obtained, m.p. 103-105 °C, ref. [S3] 105-106 °C.

3´-Benzyloxy-1,1´-biphenyl-4-carboxylic acid (**2**)

To a solution of ester **21** (5.5 g; 17.0 mmol) in dioxane (40 mL) and ethanol (20 mL), a solution of sodium hydroxide (2.4 g; 60.0 mmol) in water (25 mL) was added and the reaction mixture was heated at 80 °C for 1.5 h. The organic solvent was evaporated and to the residue conc. hydrochloric acid (6.6 mL) was added. The precipitate was filtered off and washed with water (2 × 20 mL). 5.1 g (97%) of acid **2** was obtained, m.p. 193-195 °C, ref. [S3] 185-186 °C).

1-(Benzyloxy)-4-(oct-7-en-1-yloxy)benzene (**23**)

A mixture of 8-bromooct-1-ene (4.3 g; 22 mmol), 4-benzyloxyphenol (**24**, 4.0 g; 20 mmol), potassium carbonate (5.8 g; 40 mmol) and DMF (50 mL) was stirred and heated at 65 °C for 48 h. After cooling to room temperature, the mixture was diluted with water (200 mL) and extracted with toluene (4 × 60 mL). The combined organic solution was washed with water (50 mL), brine (50 mL) and dried with anhydrous magnesium sulfate. The crude product after evaporation was purified by column chromatography (toluene) to yield 6.0 g (97%) of ether **23**, m.p. 64-65 °C. ^{1}H NMR: 1.30-1.45 (m, 6 H, $(CH_2)_3$), 1.76 (m, 2 H, $\underline{CH_2}CH_2O$), 2.06 (m, 2 H, $\underline{CH_2}CH{=}CH_2$), 3.90 (t, 2 H, J = 6.5, CH_2O), 4.97 (m, 4 H, CH_2O, $CH{=}\underline{CH_2}$), 5.79 (m, 1 H, $\underline{CH}{=}CH_2$), 6.82 (d, 2 H, J = 9.1), 6.90 (d, 2 H, J = 9.1), 7.38 (m, 5 H). Elemental analysis: for $C_{21}H_{26}O_2$ (310.43): calculated C 81.25, H 8.44; found C 81.44, H 8.39%.

1-(Benzyloxy)-4-[(9,9,10,10,11,11,12,12,12-nonafluoro-7-iodododecyl)oxy]benzene (**24**)

A mixture of AIBN (0.25 g; 1.0 mmol) and toluene (20 mL) was cooled to −70 °C in an argon atmosphere. The flask was degassed and re-filled with argon. Ether **23** (3.3 g; 11.0 mmol) and 1-iodononafluorobutane (5.2 g; 15.0 mmol) were added at room temperature and the reaction mixture was heated at 80 °C for 48 h. A new portion of AIBN (0.25 g; 1.0 mmol) and 1-iodononafluorobutane (5.2 g; 15.0 mmol) were added and the mixture was heated at 80 °C for 24 h. After cooling to room temperature, the solvent was evaporated and the crude product was purified by column chromatography (hexane/ethyl acetate 30/1) and crystallization from methanol. 5.0 g (64%) of **24** was obtained. ^{1}H NMR: 1.35-1.65 (m, 6 H, $(CH_2)_3$), 1.70-1.90 (m, 4 H, $(CH_2)_2$), 2.88 (m, 2 H, CH_2CF_2), 3.91 (t, 2 H, J = 6.5, OCH_2), 4.34 (m, 1 H, CHI), 5.02 (s, 2 H, OCH_2Ph), 6.84 (m, 2 H), 6.90 (m, 2 H), 7.37 (m, 5 H). Elemental analysis: for $C_{25}H_{26}F_9IO_2$ (656.36): calculated C 45.75, H 3.99; found C 45.55, H 3.71%.

1-(Benzyloxy)-4-[(9,9,10,10,11,11,12,12,12-nonafluorododecyl)oxy]benzene (**25**)

To a mixture of $LiAlH_4$ (0.34 g; 9.0 mmol) in dry THF (10 mL), a solution of ether **24** (3.0 g; 4.6 mmol) in dry THF (5 mL) was added drop-wise in an argon atmosphere and stirred at room temperature for 24 h. The mixture was diluted with water (50 mL) and 15% aq. sodium hydroxide solution (15 mL), and extracted with diethyl ether (3 × 60 mL). The combined organic solution was washed with water (30 mL) and dried with anhydrous magnesium

sulfate. After evaporation, the crude product was purified by crystallization from methanol to yield 2.1 g (86%) of ether **25**, m.p. 66-68 °C. ^{1}H NMR: 1.36-1.71 (m, 10 H, $(CH_2)_5$), 1.76 (m, 2 H, $OCH_2\underline{CH_2}$), 2.05 (m, 2 H, CH_2CF_2), 3.90 (t, 2 H, *J* = 6.5, OCH_2), 5.01 (s, 2 H, OCH_2Ph), 6.82 (d, 2 H, *J* = 9.1), 6.90 (d, 2 H, *J* = 9.1),7.38 (m, 5 H). Elemental analysis: for $C_{25}H_{27}F_9O_2$ (530.47): calculated C 56.60, H 5.13; found C 56.76, H 5.12%.

4-[(9,9,10,10,11,11,12,12,12-Nonafluorododecyl)oxy]phenol (**7b**)
To a solution of benzyl derivative **25** (1.4 g; 2.64 mmol) in acetone (100 mL), 10% Pd/C (0.18 g) and ammonium formate (0.67 g; 10.6 mmol) were added. The reaction mixture was heated to boiling for 4 h. The mixture was filtered while hot and the filtrate was evaporated. The crude product was purified by column chromatography (chloroform/methanol 99/1) and crystallization from hexane. Yield: 1.1 g (95%) of ester **7b**, m.p. 74-78 °C. ^{1}H NMR: 1.36-1.70 (m, 10 H, $(CH_2)_5$), 1.75 (m, 2 H, $OCH_2\underline{CH_2}$), 2.05 (m, 2 H, CH_2CF_2), 3.89 (t, 2 H, *J* = 6.5, OCH_2), 4.51 (s, 1 H, OH), 6.77 (m, 4 H). Elemental analysis: for $C_{18}H_{21}F_9O_2$ (440.34): calculated C 49.10, H 4.81; found C 48.38, H 4.73%.

(S)-4-[(tert-Butyldimethylsilyl)oxy]phenyl 2-(dodecyloxy)propanoate (**27**)
A mixture of phenol **26** (3.0 g; 13.2 mmol), (*S*)-2-dodecyloxypropanoic acid [S4] (3.0 g; 12.0 mmol), DCC (3.7 g; 18.0 mmol) and DMAP (75 mg; 0.6 mmol) in dry dichloromethane (120 mL) was stirred at room temperature for 24 h, the remaining DCC was decomposed with water (1 mL). The precipitate was filtered off, washed with dichloromethane (2 × 20 mL) and the filtrate was evaporated. The residue was chromatographed (toluene) to yield 3.5 g (63%) of oily ester **27**. ^{1}H NMR: 0.17 (s, 6 H, $Si(CH_3)_2$), 0.88 (t, 3 H, *J* = 6.4, CH_3), 0.98 (s, 9 H, $C(CH_3)_3$), 1.18-1.41 (m, 18 H, $(CH_2)_9$), 1.54 (d, 3 H, *J* = 6.7, $CH(\underline{CH_3})O$), 1.62 (m, 2 H, $OCH_2\underline{CH_2}$), 3.45 (m, 1 H, $\underline{CH_2}OCH$), 3.66 (m, 1 H, $\underline{CH_2}OCH$), 4.15 (q, 1 H, CH), 6.81 (d, 2 H, *J* = 8.8), 6.95 (d, 2 H, *J* = 8.8). Elemental analysis: for $C_{27}H_{48}O_4Si$ (464.75): calculated C 69.78, H 10.41; found C 69.91, H 10.30%.

(S)-(4-Hydroxyphenyl) 2-(dodecyloxy)propanoate (**7c**)
To a solution of ester **27** (2.8 g; 6 mmol) in THF (50 mL), 7 mL of a 1 M solution of TBAF in THF was added drop-wise at 0 °C. The reaction mixture was stirred at room temperature for 1 h, diluted with water (50 mL) and extracted with ethyl acetate (3 × 60 mL). The combined organic solution was dried with anhydrous magnesium sulfate. After evaporation, the product was purified by column chromatography (chloroform/methanol 99/1) and crystallization from hexane. 2.0 g (95%) of **7c** was obtained, m.p. 70-71 °C. ^{1}H NMR ($CDCl_3$): 0.87 (t, 3 H, *J* = 6.5, CH_3), 1.25 (m, 18 H, $(CH_2)_9$), 1.55 (d, 3 H, *J* = 6.7, $CH(\underline{CH_3})O$, 1.61 (m, 2 H, $OCH_2\underline{CH_2}$), 3.48 (m, 1 H, $\underline{CH_2}OCH$), 3.65 (m, 1 H, $\underline{CH_2}OCH$), 4.16 (q, 1 H, CH), 4.97 (s, 1 H, OH), 6.80 (d, 2 H, *J* = 8.8), 6.95 (d, 2 H, *J* = 9.1). Elemental analysis: for $C_{21}H_{34}O_4$ (350.49): calculated C 71.96, H 9.78; found C 71.83, H 9.91%.

Dodecyl 4-(4-formylbenzoyloxy)benzoate (**29**)
A mixture of dodecyl 4-hydroxybenzoate (**28**) (4.0 g; 13.1 mmol), 4-formylbenzoic acid (2.35 g; 15.7 mmol), DCC (4.8 g; 23.5 mmol) and DMAP (0.1 g; 0.78 mmol) in dry dichloromethane (150 mL) was stirred at room temperature for 24 h and then worked up as

for **27**. After column chromatography (chloroform/methanol 99/1), 4.75 g (83%) of benzoate **29** was obtained, m.p. 96-97 °C, ref. [S5] 96-97 °C.

4-[4-(Dodecyloxycarbonyl)phenoxycarbonyl]benzoic acid (**10**)
To a solution of aldehyde **29** (4.75 g; 10.8 mmol) in acetone (300 mL), Jones reagent (6 mL) was added drop wise at 0 °C and the mixture was stirred for 18 h. A new portion of Jones reagent (4 mL) was added and the mixture was stirred for further 24 h and then poured into ice water (500 mL). The precipitate was filtered off and washed with water (2 × 100 mL). Crystallization from toluene yielded 4.0 g (81%) of acid **10**, m.p. 173-228 °C. ^{1}H NMR: 0.88 (t, 3 H, *J* = 6.5, CH_3), 1.20-1.50 (m, 18 H, $(CH_2)_9$), 1.78 (m, 2 H, $OCH_2\underline{CH_2}$), 4.33 (t, 2 H, *J* = 6.5, OCH_2), 7.33 (d, 2 H, *J* = 8.8), 8.15 (d, 2 H, *J* = 8.5), 8.26 (d, 2 H, *J* = 8.8), 8.32 (d, 2 H, *J* = 8.8). Elemental analysis: for $C_{27}H_{34}O_6$ (454.56): calculated C 71.34, H 7.54; found C 71.24, H 7.48%.

3. Synthesis of the target materials I–III

4-[4´-(Methoxycarbonyloxy)-1,1´-biphenyl-3-carbonyloxy]phenyl 4-dodecyloxybenzoate (**11**)
A mixture of phenol **3** (1.24 g; 3.11 mmol), acid **1a** (0.7 g; 2.57 mmol), DCC (0.74 g; 3.58 mmol) and a catalytic amount of DMAP (50 mg) in dry dichloromethane (100 mL) was stirred at room temperature for 4 h, 0.5 mL of water was added and after 30 min stirring, the precipitate was filtered off and washed with dichloromethane (2 × 15 mL). The filtrate was evaporated and chromatographed (toluene/*tert*-butyl methyl ether 20/1). Crystallization from a toluene/hexane mixture yielded 1.01 g (61%) of **11**, m. p. 126-128 °C. ^{1}H NMR: 0.89 (t, 3 H, *J* = 6.5, CH_3), 1.25-1.60 (m, 18 H, $(CH_2)_9$), 1.83 (m, 2 H, CH_2), 3.94 (s, 3 H, CH_3OCO), 4.05 (t, 2 H, *J* = 6.5, OCH_2), 6.98 (d, 2 H, *J* = 8.8), 7.30 (m, 6 H, H-3´, H-5´), 7.60 (t, 1 H, *J* = 8.8, H-5), 7.67 (d, 2 H, *J* = 8.8, H-2´, H-6´), 7.84 (d, 1 H, *J* = 8.0, H-6), 8.15 (d, 2 H, *J* = 8.8), 8.19 (d, 1 H, *J* = 7.9, H-4), 8.41 (s, 1 H, H-2). Elemental analysis: for $C_{40}H_{44}O_8$ (652.79): calculated C 73.60, H 6.79; found C 73.38, H 6.51%.

By the same procedure, compounds **12,13** were prepared.
4-Dodecyloxyphenyl 4-[4´-(benzyloxy)-1,1´-biphenyl-3-carbonyloxy]benzoate (**12**), yield 76%, m. p. 148-150 °C. ^{1}H NMR: 0.88 (t, 3 H, *J* = 6.5, CH_3), 1.20-1.55 (m, 18 H, $(CH_2)_9$), 1.79 (m, 2 H, CH_2), 3.96 (t, 2 H, *J* = 6.5, OCH_2), 5.14 (s, 2 H, $Ph\underline{CH_2}$), 6.94 (d, 2 H, *J* = 9.1), 7.10 (m, 4 H), 7.41 (m, 7 H), 7.58 (t, 1 H, *J* = 8.8), 7.60 (d, 2 H, *J* = 8.8), 7.85 (d, 1 H, *J* = 7.6), 8.15 (d, 1 H, *J* = 7.9), 8.29 (d, 2 H, *J* = 8.5), 8.40 (s, 1 H). Elemental analysis: for $C_{45}H_{48}O_6$ (684.88): calculated C 78.92, H 7.06; found C 78.76, H 7.20%.
4-(Dodecyloxycarbonyl)phenyl 4-[4´-(benzyloxy)-1,1´-biphenyl-3-carbonyl]oxy]benzoate (**13**), yield 91%, m. p. 120-122 °C. ^{1}H NMR: 0.88 (t, 3 H, *J* = 6.5, CH_3), 1.20-1.55 (m, 18 H, $(CH_2)_9$), 1.76 (m, 2 H, CH_2), 4.33 (t, 2 H, *J* = 6.5, OCH_2), 5.14 (s, 2 H, $Ph\underline{CH_2}$), 7.09 (d, 2 H, *J* = 8.8), 7.32 (d, 2 H, *J* = 8.8), 7.41 (m, 5 H), 7.42 (d, 2 H, *J* = 8.8), 7.58 (t, 1 H, *J* = 8.8), 7.60 (d, 2 H, *J* = 8.8), 7.85 (d, 1 H, *J* = 7.6), 8.14 (m, 1 H), 8.14 (d, 2 H, *J* = 8.8), 8.31 (d, 2 H, *J* = 8.8), 8.40 (s, 1 H). Elemental analysis: for $C_{46}H_{48}O_7$ (712.89): calculated C 77.50, H 6.79; found C 77.35, H 6.93%.

4-(4´-Hydroxy-1,1´-biphenyl-3-carbonyloxy)phenyl 4-dodecyloxybenzoate (**14**)

To a solution of **11** (0.77 g; 1.18 mmol) in a mixture of ethanol (65 mL), dichloromethane (8 mL) and chloroform (22 mL), 25% aq. ammonia (8 mL) was added. The reaction mixture was stirred at room temperature for 48 h, diluted with chloroform (60 mL) and water (20 mL), cooled to 0°C and acidified with 15% aq. hydrochloric acid to pH ~2. Layers were separated, the aqueous layer was extracted with chloroform (3 × 50 mL) and the combined organic solution was dried with anhydrous magnesium sulfate. The solvent was evaporated and the crude product was purified by column chromatography (toluene/*tert*-butyl methyl ether 15/1). Yield 0.48 g (69%) of **14**, m. p. 123-125 °C. ^{1}H NMR: 0.89 (t, 3 H, *J* = 6.5, CH_3), 1.15-1.60 (m, 18 H, $(CH_2)_9$), 1.81 (m, 2 H, CH_2), 4.05 (t, 2 H, *J* = 6.5, OCH_2), 4.85 (s, 1 H, OH), 6.96 (m, 4 H, H-3´, H-5´), 7.28 (m, 4 H), 7.55 (d, 2 H, *J* = 8.8, H-2´, H-6´), 7.57 (t, 1 H, H-5), 7.82 (d, 1 H, *J* = 8.0, H-6), 8.15 (m, 3 H, H-4), 8.38 (s, 1 H, H-2). Elemental analysis: for $C_{38}H_{42}O_6$ (594.75): calculated C 76.74, H 7.12; found C 76.56, H 6.99%.

4-Dodecyloxyphenyl 4-(4´-hydroxy-1,1´-biphenyl-3-carbonyloxy)benzoate (**15**)

To a solution of benzyl derivative **12** (0.6 g; 0.88 mmol) in acetone (30 mL), 10% Pd/C (50 mg) and ammonium formate (0.2 g; 3.17 mmol) were added. The reaction mixture was heated to boiling for 2 h. The hot mixture was filtered and the filtrate was evaporated. The crude product was purified by crystallization from a acetone/hexane mixture. 0.46 g (88%) of ester **15** was obtained, m. p. 138-141 °C. ^{1}H NMR: 0.88 (t, 3 H, *J* = 6.5, CH_3), 1.25-1.60 (m, 18 H, $(CH_2)_9$), 1.78 (m, 2 H, CH_2), 3.96 (t, 2 H, *J* = 6.5, OCH_2), 4.88 (s, 1 H, OH), 6.94 (m, 4 H), 7.12 (d, 2 H, *J* = 9.0), 7.39 (d, 2 H, *J* = 8.8), 7.58 (m, 3 H), 7.83 (d, 1 H, *J* = 7.9), 8.15 (d, 1 H, *J* = 7.6), 8.29 (d, 2 H, *J* = 8.8), 8.39 (s, 1 H). Elemental analysis: for $C_{38}H_{42}O_6$ (594.75): calculated C 76.74, H 7.12; found C 76.62, H 7.03%.

By the same procedure compound **16** was prepared, yield 86%, m. p. 124-126 °C. ^{1}H NMR: 0.88 (t, 3 H, *J* = 6.5, CH_3), 1.20-1.55 (m, 18 H, $(CH_2)_9$)), 1.76 (m, 2 H , CH_2), 4.33 (t, 2 H, *J* = 6.5, OCH_2), 4.89 (s, 1 H, OH), 6.95 (d, 2 H, *J* = 8.8), 7.31 (d, 2 H, *J* = 9.1), 7.42 (d, 2 H, *J* = 9.1), 7.56 (d, 2 H, *J* = 8.8), 7.58 (t, 1 H), 7.84 (d, 1 H, *J* = 7.8), 8.14 (d, 2 H, *J* = 9.1), 8.15 (m, 1 H), 8.31 (d, 2 H, *J* = 9.1), 8.39 (s, 1 H). Elemental analysis: for $C_{39}H_{42}O_7$ (622.77): calculated C 75.22, H 6.80; found C 74.98, H 6.86%.

General procedure for synthesis of compounds **I–III**

A mixture of benzoate **14–16** (1 mmol), acid **6a–c** (1.2 mmol), DCC (1.4 mmol) and catalytic amount of DMAP in dry dichloromethane (50 mL) was stirred at room temperature in an argon atmosphere for 5 h. The precipitate was filtered off and washed with dichloromethane (2 × 10 mL). The residue after evaporation of the solvent was purified by column chromatography (toluene/*tert*-butyl methyl ether 10/1) and crystallization from ethyl acetate.

4-(4-Dodecyloxybenzoyloxy)phenyl 4´-(4-dodecyloxybenzoyloxy)-1,1´-biphenyl-3-carboxylate (**Ia**). Yield 90%. ^{1}H NMR: 0.88 (t, 6 H, *J* = 6.5, 2 × CH_3), 1.20-1.55 (m, 36 H, 2 × $(CH_2)_9$)), 1.83 (m, 4 H, 2 × $\underline{CH_2}CH_2O$), 4.05 (t, 4 H, *J* = 6.5, 2 × OCH_2), 6.98 (d, 4 H, *J* = 9.1), 7.28 (m, 4 H), 7.33 (d, 2 H, *J* = 8.8, H-3´, H-5´), 7.61 (t, 1 H, H-5), 7.71 (d, 2 H, *J* = 8.8, H-2´, H-6´),

7.87 (d, 1 H, *J* = 7.9, H-6), 8.17 (m, 5 H, H-4), 8.44 (s, 1 H, H-2). Elemental analysis: for $C_{57}H_{70}O_8$ (883.19): calculated C 77.52, H 7.99; found C 77.28, H 7.98%.

4-(4-Dodecyloxybenzoyloxy)phenyl 4´-[4-(9,9,10,10,11,11,12,12,12-nonafluorododecyloxy)-benzoyloxy]-1,1´-biphenyl-3-carboxylate (**Ib**). Yield 93%. ^{1}H NMR: 0.89 (t, 3 H, *J* = 6.5, CH_3), 1.20-1.55 (m, 26 H, $(CH_2)_9$) + $(CH_2)_4$)), 1.61 (m, 2 H), 1.83 (m, 4 H, 2 × $\underline{CH_2}CH_2O$), 2.05 (m, 2 H, CH_2CF_2), 4.05 (t, 2 H, *J* = 6.4, OCH_2), 4.06 (t, 2 H, *J* = 6.5, OCH_2), 6.98 (d, 4 H, *J* = 8.8), 7.29 (m, 4 H), 7.33 (d, 2 H, *J* = 8.5), 7.61 (t, 1 H), 7.71 (d, 2 H, *J* = 8.8), 7.87 (d, 1 H, *J* = 7.9), 8.17 (m, 5 H), 8.44 (s, 1 H). Elemental analysis: for $C_{57}H_{61}F_9O_8$ (1045.10): calculated C 65.51, H 5.88; found C 65.28, H 5.67%.

(S)-4-(4-Dodecyloxybenzoyloxy)phenyl 4´-[4-(2-dodecyloxypropanoyloxy)benzoyloxy]-1,1´-biphenyl-3-carboxylate (**Ic**). Yield 68%. ^{1}H NMR: 0.88 (m, 6 H, 2 × CH_3), 1.15-1.55 (m, 36 H, 2 ×$(CH_2)_9$)), 1.60 (d, 3 H, *J* = 6.7, $\underline{CH_3}CH$),1.61 (m, 2 H, $\underline{CH_2}CH_2O$), 1.81 (m, 2 H, $\underline{CH_2}CH_2O$), 3.51 (m, 1 H, $\underline{CH_2}OCH$), 3.70 (m, 1 H, $\underline{CH_2}OCH$), 4.05 (t, 2 H, *J* = 6.5, OCH_2), 4.22 (q, 1 H, CH), 6.98 (d, 2 H, *J* = 9.1), 7.29 (m, 6 H), 7.34 (d, 2 H, *J* = 8.8), 7.61 (t, 1 H), 7.72 (d, 2 H, *J* = 8.8), 7.88 (d, 1 H, *J* = 7.6), 8.15 (d, 2 H, *J* = 8.8), 8.20 (d, 1 H, *J* = 7.9), 8.28 (d, 2 H, *J* = 8.8), 8.44 (s, 1 H). Elemental analysis: for $C_{60}H_{74}O_{10}$ (955.25): calculated C 75.44, H 7.81; found C 75.29, H 7.98%.

4-[4-(Dodecyloxyphenoxy)carbonyl]phenyl 4´-(4-dodecyloxybenzoyloxy)-1,1´-biphenyl-3-carboxylate (**IIa**). Yield 75%. ^{1}H NMR: 0.88 (t, 6 H, *J* = 6.4, 2 × CH_3), 1.20-1.55 (m, 36 H, 2 ×$(CH_2)_9$), 1.81 (m, 4 H, 2 × $\underline{CH_2}CH_2O$), 3.96 (t, 2 H, *J* = 6.5, OCH_2), 4.05 (t, 2 H, *J* = 6.5, OCH_2), 6.94 (d, 2 H, *J* = 9.1), 6.99 (d, 2 H, *J* = 9.1), 7.13 (d, 2 H, *J* = 9.1), 7.33 (d, 2 H, *J* = 8.8), 7.40 (d, 2 H, *J* = 8.8), 7.62 (t, 1 H), 7.71 (d, 2 H, *J* = 8.8), 7.89 (d, 1 H, *J* = 7.8), 8.17 (d, 2 H, *J* = 8.8), 8.21 (d, 1 H, *J* = 7.8), 8.30 (d, 2 H, *J* = 8.8), 8.45 (s, 1 H). Elemental analysis: for $C_{57}H_{70}O_8$ (883.19): calculated C 77.52, H 7.99; found C 77.32, H 7.91%.

4-[4-(Dodecyloxyphenoxy)carbonyl]phenyl 4´-[4-(9,9,10,10,11,11,12,12,12-nonafluoro-dodecyloxy)benzoyloxy]-1,1´-biphenyl-3-carboxylate (**IIb**). Yield 85%. ^{1}H NMR: 0.88 (t, 3 H, *J* = 6.5, CH_3), 1.20-1.55 (m, 26 H, $(CH_2)_9$) + $(CH_2)_4$)), 1.61 (m, 2 H), 1.81 (m, 4 H, 2 × $\underline{CH_2}CH_2O$), 2.06 (m, 2 H, CH_2CF_2), 3.96 (t, 2 H, *J* = 6.5, OCH_2), 4.06 (t, 2 H, *J* = 6.5, OCH_2), 6.94 (d, 2 H, *J* = 9.1), 6.99 (d, 2 H, *J* = 8.8), 7.13 (d, 2 H, *J* = 9.1), 7.33 (d, 2 H, *J* = 8.5), 7.40 (d, 2 H, *J* = 8.8), 7.62 (t, 1 H), 7.71 (d, 2 H, *J* = 8.5), 7.89 (d, 1 H, *J* = 8.2), 8.17 (d, 2 H, *J* = 8.8), 8.21 (d, 1 H, *J* = 7.9), 8.30 (d, 2 H, *J* = 8.8), 8.45 (s, 1 H). Elemental analysis: for $C_{57}H_{61}F_9O_8$ (1045.10): calculated C 65.51, H 5.88; found C 65.35, H 5.62%.

(S)-4-[4-(Dodecyloxyphenoxy)carbonyl]phenyl 4´-[4-(2-dodecyloxypropanoyloxy)-benzoyloxy]-1,1´-biphenyl-3-carboxylate (**IIc**). Yield 68%. ^{1}H NMR: 0.88 (m, 6 H, 2 × CH_3), 1.20-1.55 (m, 36 H, 2 × $(CH_2)_9$), 1.60 (d, 3 H, *J* = 6.7, $\underline{CH_3}CH$), 1.65 (m, 2 H, $\underline{CH_2}CH_2O$), 1.79 (m, 2 H, $\underline{CH_2}CH_2O$), 3.50 (m, 1 H, $\underline{CH_2}OCH$), 3.70 (m, 1 H, $\underline{CH_2}OCH$), 3.96 (t, 2 H, *J* = 6.5, OCH_2), 4.22 (q, 1 H, CH), 6.94 (d, 2 H, *J* = 9.1), 7.13 (d, 2 H, *J* = 9.1), 7.29 (d, 2 H, *J* = 8.8), 7.34 (d, 2 H, *J* = 8.8), 7.40 (d, 2 H, *J* = 8.8), 7.63 (t, 1 H), 7.72 (d, 2 H, *J* = 8.8), 7.90 (d, 1 H, *J* = 8.5), 8.21 (d, 1 H, *J* = 8.2), 8.29 (m, 4 H), 8.45 (s, 1 H). Elemental analysis: for $C_{60}H_{74}O_{10}$ (955.25): calculated C 75.44, H 7.81; found C 75.31, H 7.95%.

4-[4-(Dodecyloxycarbonyl)phenoxycarbonyl]phenyl 4´-(4-dodecyloxybenzoyloxy)-1,1´-biphenyl-3-carboxylate (**IIIa**). Yield 85%. ^{1}H NMR: 0.88 (m, 6 H, 2 × CH_3), 1.20-1.55 (m, 36 H, 2 × $(CH_2)_9$), 1.80 (m, 4 H, 2 × $\underline{CH_2}CH_2O$), 4.05 (t, 2 H, *J* = 6.5, OCH_2), 4.33 (t, 2 H, *J* = 6.4, OCH_2), 6.99 (d, 2 H, *J* = 8.8), 7.33 (m, 4 H), 7.43 (d, 2 H, *J* = 8.8), 7.63 (t, 1 H), 7.71 (d,

2 H, J = 8.5), 7.90 (d, 1 H, J = 8.2), 8.15 (m, 4 H), 8.21 (d, 1 H, J = 7.9), 8.31 (d, 2 H, J = 8.8), 8.45 (s, 1 H). Elemental analysis: for $C_{58}H_{70}O_9$ (911.20): calculated C 76.45, H 7.74; found C 76.47, H 7.84%.

4-[4-(Dodecyloxycarbonyl]phenoxycarbonyl]phenyl 4´-[4-(9,9,10,10,11,11,12,12,12-nonafluorododecyloxy)benzoyloxy]-1,1´-biphenyl-3-carboxylate (**IIIb**). Yield 82%. ^{1}H NMR: 0.88 (t, 3 H, J = 6.7, CH_3), 1.20-1.55 (m, 26 H, $(CH_2)_9$ + $(CH_2)_4$)), 1.61 (m, 2 H), 1.81 (m, 4 H, 2 × $\underline{CH_2}CH_2O$), 2.06 (m, 2 H, CH_2CF_2), 4.06 (t, 2 H, J = 6.5, OCH_2), 4.33 (t, 2 H, J = 6.5, OCH_2), 6.99 (d, 2 H, J = 9.1), 7.33 (m, 4 H), 7.43 (d, 2 H, J = 8.5), 7.63 (t, 1 H), 7.71 (d, 2 H, J = 8.5), 7.90 (d, 1 H, J = 8.2), 8.15 (m, 4 H), 8.21 (d, 1 H, J = 7.9), 8.31 (d, 2 H, J = 8.5), 8.45 (s, 1 H). Elemental analysis: for $C_{58}H_{61}F_9O_9$ (1073.11): calculated C 64.92, H 5.73; found C 64.71, H 5.82%.

(S)-4-[4-(Dodecyloxycarbonyl)phenoxycarbonyl]phenyl 4´-[4-(2-dodecyloxypropanoyloxy)-benzoyloxy]-1,1´-biphenyl-3-carboxylate (**IIIc**). Yield 54%. ^{1}H NMR: 0.88 (t, 6 H, 2 × CH_3), 1.20-1.55 (m, 36 H, 2 × $(CH_2)_9$), 1.60 (d, 3 H, J = 6.7, $\underline{CH_3}CH$),1.64 (m, 2 H, $\underline{CH_2}CH_2O$), 1.80 (m, 2 H, $\underline{CH_2}CH_2O$), 3.51 (m, 1 H, $\underline{CH_2}OCH$), 3.70 (m, 1 H, $\underline{CH_2}OCH$), 4.33 (t, 2 H, J = 6.5, OCH_2), 4.22 (q, 1 H, CH), 7.31 (m, 6 H), 7.43 (d, 2 H, J = 8.8), 7.63 (t, 1 H), 7.72 (d, 2 H, J = 8.8), 7.90 (d, 1 H, J = 6.5), 8.14 (d, 2 H, J = 8.8), 8.22 (d, 1 H, J = 6.6), 8.28 (d, 2 H, J = 9.5), 8.31 (d, 2 H, J = 9.1), 8.45 (s, 1 H). Elemental analysis: for $C_{61}H_{74}O_{11}$ (983.26): calculated C 74.52, H 7.59; found C 74.30, H 7.76%.

4. Synthesis of target materials IV–VI

4-(Dodecyloxy)phenyl 3´-benzyloxy-1,1´-biphenyl-4-carboxylate (**17a**)

A mixture of acid **2** (2.0 g; 6.6 mmol), phenol **7a** (1.93 g; 6.93 mmol), DCC (2.04 g; 9.9 mmol) and catalytic amount of DMAP (25 mg) in dry dichloromethane (200 mL) was stirred at room temperature in an argon atmosphere for 12 h. The precipitate was filtered off and washed with dichloromethane (2 × 20 mL). The residue after evaporation of the solvent was purified by column chromatography (chloroform/methanol 99/1). Yield 2.3 g (68%) of ester **17a**, m.p. 77-79 °C. ^{1}H NMR: 0.88 (t, 3 H, J = 6.4, CH_3), 1.18-1.55 (m, 18 H, $(CH_2)_9$), 1.78 (m, 2 H, $OCH_2\underline{CH_2}$), 3.96 (t, 2 H, J = 6.5, OCH_2), 5.15 (s, 2 H, $O\underline{CH_2}Ph$), 6.94 (d, 2 H, J = 9.1), 7.03 (m, 1 H), 7.13 (d, 2 H, J = 9.1), 7.41 (m, 8 H), 7.71 (d, 2 H, J = 8.5), 8.25 (d, 2 H, J = 8.8). Elemental analysis: for $C_{38}H_{44}O_4$ (564.75): calculated C 80.82, H 7.85; C 80.71, H 7.78%.

By the same procedure, compounds **17b,c** were prepared by the reaction of **2** with phenol **7b,c**, resp.

4-(9,9,10,10,11,11,12,12,12-Nonafluorododecyloxy)phenyl 3´-benzyloxy-1,1´-biphenyl-4-carboxylate (**17b**). Yield 71%, m.p. 86-89 °C. ^{1}H NMR: 1.30-1.50 (m, 10 H, $(CH_2)_5$), 1.80 (m, 2 H, $OCH_2\underline{CH_2}$), 2.05 (m, 2 H, CH_2CF_2), 3.97 (t, 2 H, J = 6.5, $OCH_2\underline{CH_2}$), 5.15 (s, 2 H, OCH_2Ph), 6.93 (d, 2 H, J = 8.8), 7.03 (m, 1 H), 7.13 (d, 2 H, J = 8.8), 7.40 (m, 8 H), 7.70 (d, 2 H, J = 8.2), 8.25 (d, 2 H, J = 8.2). Elemental analysis: for $C_{38}H_{35}F_9O_4$ (726.67): calculated C 62.81, H 4.85; found C 62.93, H 4.81%.

(*S*)*-4-[2-(Dodecyloxy)propanoyloxy]phenyl 3´-benzyloxy-1,1´-biphenyl-4-carboxylate* (**17c**). Yield 55%, m.p. 80-81 °C. ^{1}H NMR: 0.88 (t, 3 H, *J* = 6.5, CH_3), 1.20-1.41 (m, 18 H, $(CH_2)_9$), 1.58 (d, 3 H, *J* = 6.7; CH_3CH)), 1.65 (m, 2 H, OCH_2CH_2), 3.49 (m, 1 H, CH_2OCH), 3.68 (m, 1 H, CH_2OCH), 4.16 (q, 1 H, CH), 5.15 (s, 2 H, OCH_2Ph), 7.03 (m, 1 H), 7.18 (m, 2 H), 7.27 (m, 4 H), 7.41 (m, 6 H), 7.71 (d, 2 H, *J* = 8.8), 8.25 (d, 2 H, *J* = 8.8). Elemental analysis: for $C_{41}H_{48}O_6$ (636.82): calculated C 77.33, H 7.60; found C 77.12, H 7.51%.

4-(Dodecyloxy)phenyl 3´-hydroxy-1,1´-biphenyl-4-carboxylate (**18a**)

To a solution of benzyl derivative **17a** (2.1 g; 3.7 mmol) in acetone (100 mL), 10% Pd/C (180 mg) and ammonium formate (0.88 g; 13.92 mmol) were added. The reaction mixture was heated to boiling for 4 h. The hot mixture was filtered and the filtrate was evaporated. The crude product was purified by crystallization from acetone/hexane mixture. 1.5 g (86%) of ester **18a** was obtained, m.p. 132-140 °C. ^{1}H NMR: 0.88 (t, 3 H, *J* = 6.7, CH_3), 1.18-1.55 (m, 18 H, $(CH_2)_9$), 1.80 (m, 2 H, OCH_2CH_2), 3.96 (t, 2 H, *J* = 6.5, OCH_2), 5.01 (s, 1 H, OH), 6.88 (m, 1 H), 6.94 (d, 2 H, *J* = 9.1), 7.13 (m, 3 H), 7.23 (m, 1 H), 7.35 (t, 1 H), 7.70 (d, 2 H, *J* = 8.5), 8.25 (d, 2 H, *J* = 8.5). Elemental analysis: for $C_{31}H_{38}O_4$ (474.63): calculated C 78.45, H 8.07; found C 78.29, H 8.13%.

By the same procedure, compounds **18b,c** were prepared from **17b,c**.

4-(9,9,10,10,11,11,12,12,12-Nonafluorododecyloxy)phenyl 3´-hydroxy-1,1´-biphenyl-4-carboxylate (**18b**), yield 94%, m.p. 126-190 °C. ^{1}H NMR: 1.30-1.44 (m, 10 H, $(CH_2)_5$), 1.80 (m, 2 H, CH_2CH_2O), 2.05 (m, 2 H, CH_2CF_2), 3.97 (t, 2 H, *J* = 6.5, CH_2O), 4.85 (s, 1 H, OH), 6.91 (m, 1 H), 6.93 (d, 2 H, *J* = 9.1), 7.13 (m, 3 H), 7.23 (m, 1 H), 7.33 (t, 1 H), 7.70 (d, 2 H, *J* = 8.2), 8.25 (d, 2 H, *J* = 8.5). Elemental analysis: for $C_{31}H_{29}F_9O_4$ (636.55): calculated C 58.49, H 4.59; found C 58.70, H 4.60%.

(S)-4-[2-(Dodecyloxypropanoyloxy)phenyl] 3´-hydroxy-1,1´-biphenyl-4-carboxylate **(18c)**, yield 95%, m.p. 87-112 °C. ^{1}H NMR: 0.88 (t, 3 H, *J* = 6.5, CH_3), 1.20-1.41 (m, 18 H, $(CH_2)_9$), 1.58 (d, 3 H, *J* = 7.6, CH_3CH), 1.66 (m, 2 H, OCH_2CH_2), 3.49 (m, 1 H, CH_2OCH),□ 3.68 (m, 1 H, CH_2OCH), 4.20 (q, 1 H, CH), 4.99 (s, 1 H, OH), 6.89 (m, 1 H), 7.13 (m, 1 H), 7.19 (m, 3 H), 7.26 (m, 2 H), 7.36 (t, 1 H), 7.71 (d, 2 H, *J* = 8.2), 8.25 (d, 2 H, *J* = 8.5). Elemental analysis for $C_{34}H_{42}O_6$ (546.69): calculated C 74.70, H 7.74; found C 74.59, H 7.92%.

General procedure for synthesis of compounds **IV-VI**

A mixture of phenol **18a-c** (1.0 mmol), acid **8-10** (1.0 mmol), DCC (1.5 mmol) and catalytic amount of DMAP (25 mg) in dry dichloromethane (50 mL) was stirred at room temperature in an argon atmosphere for 5 h. The precipitate was filtered off and washed with dichloromethane (2 × 10 mL). The solvent was evaporated and the product was purified by column chromatography (chloroform/methanol 99/1) and crystallization from ethyl acetate.

4-Dodecyloxyphenyl 3´-[4-(4-dodecyloxybenzoyloxy)benzoyloxy]-1,1´-biphenyl-4-carboxylate (**IVa**). Yield 79%. ^{1}H NMR: 0.89 (t, 6 H, *J* = 6.5, 2 × CH_3), 1.21-1.55 (m, 36 H, 2 × $(CH_2)_9$), 1.80 (m, 4 H, 2 × OCH_2CH_2), 3.96 (t, 2 H, *J* = 6.5, OCH_2), 4.06 (t, 2 H, *J* = 6.7, OCH_2), 6.94 (d, 2 H, *J* = 8.8), 6.99 (d, 2 H, *J* = 8.5), 7.14 (d, 2 H, *J* = 8.8), 7.28 (m, 1 H), 7.39 (d, 2 H, *J* =

8.5), 7.55 (m, 3 H), 7.75 (d, 2 H, *J* = 8.2), 8.16 (d, 2 H, *J* = 8.8), 8.27 (d, 2 H, *J* = 8.2), 8.31 (d, 2 H, *J* = 8.5).Elemental analysis: for $C_{57}H_{70}O_8$ (883.16): calculated C 77.52, H 7.99; found C 77.30, H 8.08%.

4-[(9,9,10,10,11,11,12,12,12-Nonafluorododecyl)oxy]phenyl 3´-[4-(4-dodecyloxybenzoyloxy)-benzoyloxy]-1,1´-biphenyl-4-carboxylate (**IVb**). Yield 59%. ^{1}H NMR: 0.88 (t, 3 H, *J* = 6.5, CH_3), 1.25-1.62 (m, 28 H, $(CH_2)_9$ + $(CH_2)_5$), 1.80 (m, 4 H, 2 × $OCH_2\underline{CH_2}$), 2.05 (m, 2 H, CH_2CF_2), 3.97 (t, 2 H, *J* = 6.7, CH_2O), 4.06 (t, 2 H, *J* = 6.7, CH_2O), 6.94 (d, 2 H, *J* = 9.1), 6.99 (d, 2 H, *J* = 8.8), 7.14 (d, 2 H, *J* = 9.1), 7.28 (m, 1 H), 7.39 (d, 2 H, *J* = 8.5), 7.55 (m, 3 H), 7.75 (d, 2 H, *J* = 8.5), 8.16 (d, 2 H, *J* = 8.5), 8.27 (d, 2 H, *J* = 8.5), 8.31 (d, 2 H, *J* = 8.5). Elemental analysis: for $C_{57}H_{61}F_9O_8$ (1045.08): calculated C 65.51, H 5.88; found C 65.34, H 5.75%.

(S)-[4-(2-Dodecyloxypropanoyloxy)phenyl] 3´-[4-(4-dodecyloxybenzoyloxy)benzoyloxy]-1,1´-biphenyl-4-carboxylate (**IVc**). Yield 62%. ^{1}H NMR: 0.88 (m, 6 H, 2 × CH_3), 1.22-1.51 (m, 36 H, 2 × $(CH_2)_9$), 1.58 (d, 3 H, *J* = 6.7, $\underline{CH_3}CH$)), 1.66 (m, 2 H, $OCH_2\underline{CH_2}$), 1.84 (m, 2 H, $OCH_2\underline{CH_2}$), 3.49 (m, 1 H, $\underline{CH_2}OCH$), 3.68 (m, 1 H, $\underline{CH_2}OCH$), 4.06 (t, 2 H, *J* = 6.7, OCH_2) 4.20 (q, 1 H, CH), 6.99 (d, 2 H, *J* = 9.1), 7.19 (d, 2 H, *J* = 9.1), 7.29 (m, 3 H), 7.40 (d, 2 H, *J* = 8.5), 7.55 (m, 3 H), 7.76 (d, 2 H, *J* = 8.2), 8.16 (d, 2 H, *J* = 8.8), 8.27 (d, 2 H, *J* = 8.2), 8.31 (d, 2 H, *J* = 8.8). Elemental analysis for $C_{60}H_{74}O_{10}$ (955.22): calculated C 75.44, H 7.81; found C 75.34, H 7.78%.

4-Dodecyloxyphenyl 3´-[4-(4-dodecyloxyphenoxycarbonyl)benzoyloxy]-1,1´-biphenyl-4-carboxylate (**Va**). Yield 61%. ^{1}H NMR: 0.88 (t, 6 H, *J* = 6.5, 2 × CH_3), 1.21-1.55 (m, 36 H, 2 × $(CH_2)_9$), 1.80 (m, 4 H, 2 × ($OCH_2\underline{CH_2}$)), 3.97 (m, 4 H, 2 × OCH_2), 6.94 (m, 4 H), 7.14 (m, 4 H), 7.30 (m, 1 H), 7.57 (m, 3 H), 7.75 (d, 2 H, *J* = 8.2), 8.28 (d, 2 H, *J* = 8.5), 8.35 (s, 4 H). Elemental analysis: for $C_{57}H_{70}O_8$ (883.16): calculated C 77.52, H 7.99; found C 77.38, H 7.91%.

4-[(9,9,10,10,11,11,12,12,12-Nonafluorododecyl)oxy]phenyl 3´-[4-(4-dodecyloxyphenoxy-carbonyl)benzoyloxy]-1,1´-biphenyl-4-carboxylate (**Vb**). Yield 70%. ^{1}H NMR: 0.88 (t, 3 H, *J* = 6.5, CH_3), 1.20-1.65 (m, 28 H, $(CH_2)_9$ + $(CH_2)_5$), 1.80 (m, 4 H, 2 × $\underline{CH_2}CH_2O$), 2.05 (m, 2 H, CH_2CF_2), 3.97 (t, 4 H, *J* = 6.5, 2 × CH_2O), 6.94 (m, 4 H), 7.14 (m, 4 H), 7.31 (m, 1 H), 7.57 (m, 3 H), 7.75 (d, 2 H, *J* = 8.2), 8.27 (d, 2 H, *J* = 8.5), 8.35 (s, 4 H). Elemental analysis: for $C_{57}H_{61}F_9O_8$ (1045.08): calculated C 65.51, H 5.88; found C 65.39, H 5.78%.

(S)-[4-(2-Dodecyloxypropanoyloxy)phenyl] 3´-[4-(4-dodecyloxyphenoxycarbonyl)benzoyl-oxy]-1,1´-biphenyl-4-carboxylate (**Vc**). Yield 59%. ^{1}H NMR: 0.89 (m, 6 H, 2 × CH_3), 1.22-1.51 (m, 36 H, 2 × $(CH_2)_9$), 1.58 (d, 3 H, *J* = 6.7, $\underline{CH_3}CH$), 1.66 (m, 2 H, $OCH_2\underline{CH_2}$), 1.80 (m, 2 H, $OCH_2\underline{CH_2}$), 3.49 (m, 1 H, $\underline{CH_2}OCH$), 3.68 (m, 1 H, $\underline{CH_2}OCH$), 3.97 (t, 2 H, *J* = 6.7, OCH_2), 4.20 (q, 1 H, CH), 6.95 (d, 2 H, *J* = 9.1), 7.15 (d, 2 H, *J* = 9.1), 7.19 (d, 2 H, *J* = 9.3), 7.29 (m, 3 H), 7.58 (m, 3 H), 7.76 (d, 2 H, *J* = 8.5), 8.27 (d, 2 H, *J* = 8.2), 8.35 (s, 4 H). Elemental analysis: for $C_{60}H_{74}O_{10}$ (955.22): calculated C 75.44, H 7.81; found C 75.26, H 7.73%.

4-Dodecyloxyphenyl 3´-{4-[(4-dodecyloxycarbonyl)phenoxycarbonyl]benzoyloxy}-1,1´-biphenyl-4-carboxylate (**VIa**). Yield 53%. ^{1}H NMR: 0.89 (t, 6 H, *J* = 6.5, 2 × CH_3), 1.21-1.55 (m, 36 H, 2 × $(CH_2)_9$), 1.80 (m, 4 H, 2 × $OCH_2\underline{CH_2}$), 3.96 (t, 2 H, *J* = 6.7, OCH_2), 4.34 (t, 2 H, *J* = 6.7, $COOCH_2$), 6.94 (d, 2 H, *J* = 9.1), 7.13 (d, 2 H, *J* = 9.1), 7.32 (m, 3 H), 7.57 (m, 3 H), 7.75 (d, 2 H, *J* = 8.5), 8.16 (d, 2 H, *J* = 8.8), 8.27 (d, 2 H, *J* = 8.5), 8.37 (m, 4 H).

Elemental analysis: for $C_{58}H_{70}O_9$ (911.17): calculated C 76.45, H 7.74; found C 76.31, H 7.82%.

4-[(9,9,10,10,11,11,12,12,12-Nonafluorododecyl)oxy]phenyl 3´-{4-[(4-dodecyloxycarbonyl)-phenoxycarbonyl]benzoyloxy}-1,1´-biphenyl-4-carboxylate (**VIb**). Yield 63%. ^{1}H NMR: 0.88 (t, 3 H, *J* = 6.5, CH_3), 1.20-1.60 (m, 28 H, $(CH_2)_9$ + $(CH_2)_5$), 1.80 (m, 4 H, 2 × $\underline{CH_2}CH_2O$), 2.05 (m, 2 H, CH_2CF_2), 3.97 (t, 2 H, *J* = 6.7, CH_2O), 4.34 (t, 2 H, *J* = 6.5, $COOCH_2$), 6.94 (d, 2 H, *J* = 9.1), 7.14 (d, 2 H, *J* = 9.1),7. 30 (m, 1 H), 7.34 (d, 2 H, *J* = 8.8), 7.58 (m, 3 H), 7.75 (d, 2 H, *J* = 8.5), 8.16 (d, 2 H, *J* = 8.5), 8.27 (d, 2 H, *J* = 8.2), 8.37 (m, 4 H). Elemental analysis: for $C_{58}H_{61}F_9O_9$ (1073.09): calculated C 64.92, H 5.73; found C 64.88, H 5.62%.

(*S*)*-[4-(2-Dodecyloxypropanoyloxy)phenyl] 3´-{4-[(4-dodecyloxycarbonyl)phenoxy-carbonyl]benzoyloxy}-1,1´-biphenyl-4-carboxylate* (**VIc**). Yield 63%. ^{1}H NMR: 0.88 (m, 6 H, 2 × CH_3), 1.20-1.51 (m, 36 H, 2 × $(CH_2)_9$), 1.58 (d, 3 H, *J* = 6.7, $\underline{CH_3}CH$), 1.66 (m, 2 H, $OCH_2\underline{CH_2}$), 1.79 (m, 2 H, $OCH_2\underline{CH_2}$), 3.49 (m, 1 H, $\underline{CH_2}OCH$), 3.68 (m, 1 H, $\underline{CH_2}OCH$), 4.20 (q, 1 H, CH), 4.34 (t, 2 H, *J* = 6.7, OCH_2), 7.19 (d, 2 H, *J* = 9.3), 7.29 (m, 3 H), 7.34 (d, 2 H, *J* = 8.8), 7.58 (m, 3 H), 7.76 (d, 2 H, *J* = 8.5), 8.16 (d, 2 H, *J* = 8.5), 8.28 (d, 2 H, *J* = 8.5), 8.37 (m, 4 H). Elemental analysis: for $C_{61}H_{74}O_{11}$ (983.23): calculated C 74.51, H 7.59; found C 74.38, H 7.64%.

5. Results

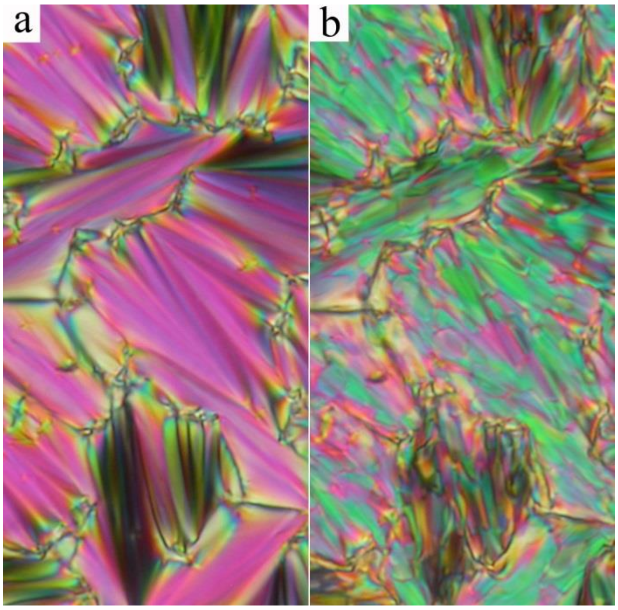

Figure S1: Microphotographs of the planar texture of **IIIb** in a) the SmAP and b) the B_{1Rev} phase. The width of both photos is about 200 μm.

6. References

S1. Kohout, M.; Svoboda, J.; Novotná, V.; Pociecha, D.; Glogarová, M.; Gorecka, E. *J. Mater. Chem.*, **2009**, *19*, 3153-3160.

S2. Dauben, W. G.; Tanabe, M. *J. Am. Chem. Soc.*, **1953**, *75*, 4969-4973.

S3. Tilley, J. W.; Clader, J. W.; Zawoiski, S.; Wirkus, M.; LeMathieu, R. A.; O'Donnell, M.; Cowley, H.; Welton, A. F. *J. Med. Chem.*, **1989**, *32*, 1814-1820.

S4. Černovská, K.; Košata, B.; Svoboda, J.; Novotná, V.; Glogarová, M. *Liq. Cryst.*, **2006**, *33*, 987-996.

S5. Haramoto, Y.; Kamogawa, H. *Mol. Cryst. Liq. Cryst.*, **1991**, *201*, 161-166.
